# Structure of Gcn1 bound to stalled and colliding 80S ribosomes

**DOI:** 10.1073/pnas.2022756118

**Published:** 2021-03-31

**Authors:** Agnieszka A. Pochopien, Bertrand Beckert, Sergo Kasvandik, Otto Berninghausen, Roland Beckmann, Tanel Tenson, Daniel N. Wilson

**Affiliations:** ^a^Institute for Biochemistry and Molecular Biology, University of Hamburg, 20146 Hamburg, Germany;; ^b^Institute of Technology, University of Tartu, 50411 Tartu, Estonia;; ^c^Gene Center, University of Munich, 81377 Munich, Germany;; ^d^Department of Biochemistry, University of Munich, 81377 Munich, Germany

**Keywords:** disome, Gcn1, ribosome, stress, translation

## Abstract

The Gcn pathway is conserved in all eukaryotes, including mammals such as humans, where it is a crucial part of the integrated stress response (ISR). Gcn1 serves as an essential effector protein for the kinase Gcn2, which in turn is activated by stalled ribosomes, leading to phosphorylation of eIF2 and a subsequent global repression of translation. The fine-tuning of this adaptive response is performed by the Rbg2/Gir2 complex, a negative regulator of Gcn2. Despite the wealth of available biochemical data, information on structures of Gcn proteins on the ribosome has remained elusive. Here we present a cryo-electron microscopy structure of the yeast Gcn1 protein in complex with stalled and colliding 80S ribosomes. Gcn1 interacts with both 80S ribosomes within the disome, such that the Gcn1 HEAT repeats span from the P-stalk region on the colliding ribosome to the P-stalk and the A-site region of the lead ribosome. The lead ribosome is stalled in a nonrotated state with peptidyl-tRNA in the A-site, uncharged tRNA in the P-site, eIF5A in the E-site, and Rbg2/Gir2 in the A-site factor binding region. By contrast, the colliding ribosome adopts a rotated state with peptidyl-tRNA in a hybrid A/P-site, uncharged-tRNA in the P/E-site, and Mbf1 bound adjacent to the mRNA entry channel on the 40S subunit. Collectively, our findings reveal the interaction mode of the Gcn2-activating protein Gcn1 with colliding ribosomes and provide insight into the regulation of Gcn2 activation. The binding of Gcn1 to a disome has important implications not only for the Gcn2-activated ISR, but also for the general ribosome-associated quality control pathways.

## Significance

There is growing evidence that collisions between ribosomes represent a cellular signal for activating multiple stress pathways, such as ribosome-associated quality control (RQC), the ribotoxic stress response, and the integrated stress response (ISR). Here we illustrate how a single protein can monitor both ribosomes within a disome, by presenting a cryo-electron microscopy structure of a native complex of the ISR protein Gcn1 interacting with both the leading stalled ribosome and the following colliding ribosome. The structure provides insight into the regulation of Gcn2 activation in yeast and has implications for the interplay between the RQC and ISR pathways in eukaryotic cells.

All living cells must adapt to a variety of different environmental stresses in a rapid and efficient way to survive. Critical to this adaptation is the integrated stress response (ISR), a central signaling network that enables cells to maintain cellular homeostasis or enter into apoptosis. In metazoans, the ISR comprises four different kinases that each phosphorylate serine 51 of the alpha subunit of eukaryotic initiation factor 2 (eIF2). In yeast and mammalian cells, the ancestral Gcn2 (general control nonderepressible 2) kinase modulates the response to nutrient deprivation (1, 2). In mammals, Gcn2 is important for long-term memory formation, feeding behavior, and immune system regulation and also has been implicated in various diseases, including neurologic disorders such as Alzheimer’s, cancers, and viral infections (1, 2). Although phosphorylation of eIF2 causes a global repression of translation initiation, translations of specific mRNAs also become up-regulated, such as the transcriptional regulator Gcn4 (yeast) or ATF4 (mammals). This in turn induces expression of genes, such as those involved in amino acid biosynthesis, to counteract the amino acid deficiency.

**Fig. 1.**
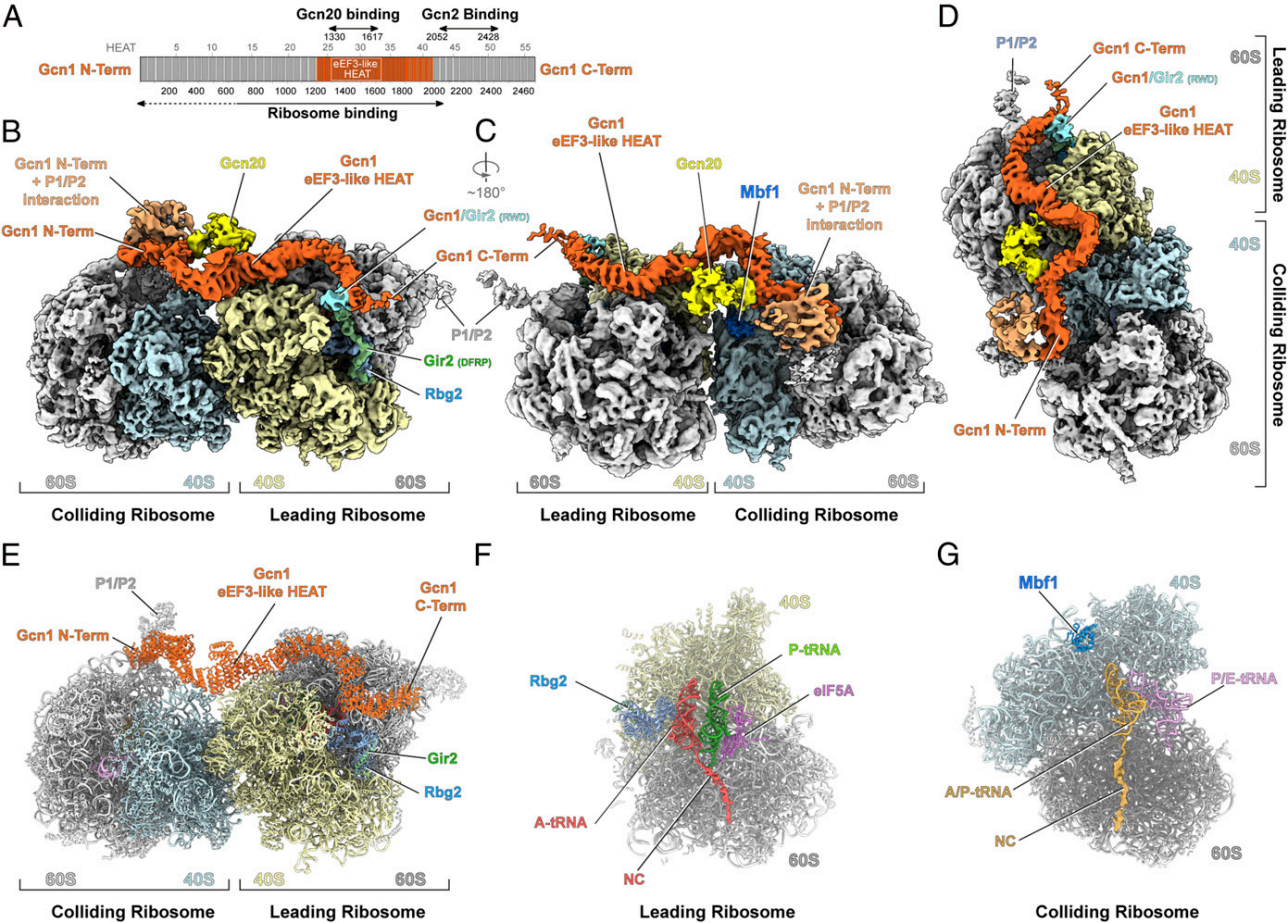
The structure of the Gcn1-bound disome. (*A*) Schematic representation of the yeast Gcn1 protein with its predicted HEAT repeats (gray boxes). Areas of Gcn1 binding to the ribosome, Gcn20, and Gcn2 are indicated with arrows. (*B*–*D*) Cryo-EM reconstruction of the Gcn1-disome complex with segmented densities for Gcn1 (orange) and Gcn20 (yellow). On the leading ribosome, 40S (cyan), 60S (gray), Rbg2 (light blue), Gir2 (green), and Gir2-RWD (cyan); on the colliding ribosome, 40S (pale yellow), 60S (gray), Mbf1 (deep blue), and the Gcn1 interaction with P1/P2-stalk proteins (salmon). (*E*–*G*) Molecular models of the Gcn1-bound disome structure (*E*); a cut-through view of the Gcn1-disome leading ribosome with Rbg2, peptidyl-tRNA (red) in the A-site, deacylated tRNA (green) in the P-site, and eIF5A (purple) in the E-site (*F*); and the colliding ribosome with Mbf1 (dark blue), peptidyl-tRNA (gold) in the A/P-site, and deacylated tRNA (purple) in the P/E-site (*G*).

The prevailing model for Gcn2 activation during nutrient deprivation is that Gcn2 recognizes and binds ribosomes that have become stalled during translation due to the accumulation of uncharged (deacylated) transfer RNAs (tRNAs) binding to the ribosomal A-site (1, 2). The activation of Gcn2 strictly requires its coactivator Gcn1 (3), a large protein (2,672 amino acids, or 297 kDa in yeast) conserved from yeast to humans (2). Based on secondary structure predictions, Gcn1 is composed almost entirely of HEAT repeats (Fig. 1*A*). The N-terminal three-quarters (residues 1 to 2052) of Gcn1 are required for tight association with ribosomes in vivo (4) (Fig. 1*A*), and a reduction in ribosome binding of Gcn1 leads to a concomitant loss in Gcn2 activation (5). The central region of Gcn1 is highly homologous to the N-terminal HEAT repeat region of the eukaryotic elongation factor 3 (eEF3) (3) (Fig. 1*A*), and overexpression of eEF3 represses Gcn2 activity, suggesting that Gcn1 and eEF3 have overlapping binding sites on the ribosome (6).

The eEF3-like region of Gcn1 is also important for interaction with the N terminus of Gcn20 (7, 8) (Fig. 1*A*), a nonessential ATP-binding cassette (ABC) protein that enhances Gcn2 activity (8). Gcn20 itself does not interact with the ribosome or with Gcn2, suggesting that Gcn20 exerts its stimulatory effect on Gcn2 via interaction and stabilization of Gcn1 on the ribosome (8). By contrast, a region (residues 2052 to 2428) within the C terminus of Gcn1 mediates direct interaction with the N-terminal RWD domain of Gcn2 (Fig. 1*A*) (4, 9, 10). Moreover, mutations within these regions of either Gcn1 (F2291L or R2259A) or Gcn2 (Y74A) disrupt the Gcn1–Gcn2 interaction, resulting in loss of both eIF2 phosphorylation and derepression of Gcn4 translation (4, 10).

Like Gcn2, Gir2 (Genetically interacts with ribosomal genes 2) contains an N-terminal RWD domain and interacts with Gcn1 (11). Overexpression of Gir2 prevents Gcn2 activation by competing with Gcn2 for Gcn1 binding (11). Gir2 forms a complex with the ribosome binding GTPase 2 (Rbg2) (12, 13), which has been proposed to dampen the Gcn2 response (13). Despite the high conservation and importance of the Gcn pathway, as well as decades of research into the Gcn proteins (1, 2), a structural basis for their mechanism of action on the ribosome has been lacking.

## Results

### Cryo-Electron Microscopy Structure of a Native Gcn1-Disome Complex.

To investigate how Gcn proteins interact with the ribosome, we set out to determine a cryo-electron microscopy (EM) structure of a Gcn-ribosome complex. To obtain such a complex, we used affinity chromatography in combination with *Saccharomyces cerevisiae* cells expressing chromosomally TAP-tagged Gcn20. A C-terminal tag was favored since the N terminus of Gcn20 is required for Gcn1 interaction (7, 8), and C-terminally tagged Gcn20 was previously shown to be indistinguishable from wild-type in complementing Δ*gcn20* deletion strains (8). Gcn1 is reported to interact with translating ribosomes in both the presence and absence of amino acid starvation (7); therefore, purification was performed with and without 3-amino-1,2,4-tirazole (3-AT), which induces histidine starvation and leads to a loss of polysomes via phosphorylation and inactivation of eIF2 (*SI Appendix*, Fig. S1 *A*–*D*). In both cases, copurification of Gcn1 and ribosomal proteins with Gcn20-TAP was observed (*SI Appendix*, Fig. S1 *B* and *C*) and validated by mass spectrometry (Dataset S1). The Gcn20-TAP eluate underwent a mild glutaraldehyde cross-linking treatment before being applied to cryo-grids and subjected to multiparticle cryo-EM. Despite repeated attempts, we were not able to visualize Gcn1 on the ribosomes from samples treated with 3-AT, whereas a low- resolution cryo-EM reconstruction of the untreated Gcn20-TAP sample revealed that a minor (5%) subpopulation of ribosomes contained an additional tube-like density, which we assigned to Gcn1 (*SI Appendix*, Fig. S2). Interestingly, this class also contained extra density located on the solvent side of the small 40S subunit, which after refinement (to 21 Å) using a larger box size, revealed that Gcn1 was associated with a disome (two 80S ribosomes) rather than a single 80S monosome (*SI Appendix*, Fig. S2). These findings suggest that if Gcn1 can interact with monosomes, the binding appears to be more labile than that to a disome.

To improve the resolution of the Gcn1-disome complex, we collected 16,823 micrographs on a Titan Krios transmission electron microscope with a Falcon II direct electron detector. Following two-dimensional classification, the remaining 616,079 ribosomal particles were subjected to three-dimensional classification and divided into 15 different classes (*SI Appendix*, Fig. S3). A diverse range of ribosome functional states that did not contain Gcn1 were identified, most of which are likely to have copurified with the polysomes to which the Gcn1-Gcn20-disome was bound. Since many of the states have been previously reported, they will not be discussed further, with the exception of class 5, which contained a posttranslocational (P- and E-site tRNAs) state ribosome with eRF1 and eEF3 present (*SI Appendix*, Fig. S3). eEF3 has been previously reported to facilitate E-site tRNA release during elongation (14); however, our results suggest that eEF3 may also perform an analogous function during translation termination. Moreover, since the eEF3-binding site overlaps with Gcn1, and the previous eEF3-ribosome structure was at 9.9 Å (15), we refined the eRF1-eEF3-ribosome structure to an average resolution of 4.2 Å. Two of the 15 classes contained density that we attributed to Gcn1 (1, 2) (*SI Appendix*, Fig. S3) based on overlap with the eEF3-binding site on the ribosome as well as the tube-like density feature characteristic of linear solenoid HEAT repeat proteins (16).

### Gcn1 Interacts with Both the Leading Stalled and Colliding Ribosomes.

Through further subsorting and local refinement (*SI Appendix*, Fig. S3), we could obtain a cryo-EM structure of the complete Gcn1-disome with an average resolution of 4.0 Å for the leading stalled ribosome; however, the colliding ribosome was poorly resolved (8.4 Å), indicating some flexibility with respect to the leading ribosome (*SI Appendix*, Fig. S4 *A*–*C*). Thus, we implemented focused refinement of the individual leading and colliding ribosomes, yielding average resolutions of 3.9 Å and 4.4 Å, respectively (*SI Appendix*, Fig. S4 *D*–*K* and Table S1). These maps were combined to generate a cryo-EM map of the complete disome, revealing how density for Gcn1 snakes its way along the disome and fuses at each end with density for the P-stalk proteins of both ribosomes (Fig. 1 *B*–*D* and Movie S1). We attributed the extra density contacting Gcn1 at the interface between the leading and colliding ribosomes to the N-terminal domain of Gcn20 (Fig. 1 *B*–*D*), since this region of Gcn1 is critical for interaction with the N terminus of Gcn20 (7, 8). However, the density is poorly resolved, and thus no model could be built for this region. In addition to Gcn1, we observed density for Rbg2/Gir2 in the A-site of the leading ribosome, as well as multiprotein bridging factor 1 (Mbf1) on the 40S subunit of the colliding ribosome (Fig. 1 *B*–*D* and Movie S1), the details and implications of which are discussed below.

To improve the density for Gcn1, an additional focused refinement was performed using a mask encompassing Gcn1 and the 40S head of the leading ribosome (*SI Appendix*, Fig. S4 *L*–*O*). The local resolution of Gcn1 was highest (4 to 7 Å) for the central eEF3-like region of Gcn1 and progressively decreased toward the N- and C-terminal ends (*SI Appendix*, Fig. S4 *L*–*O*). A molecular model for the central region of Gcn1 could be generated based on homology with eEF3 (*SI Appendix*, Fig. S5 *A*–*E*), and individual HEAT repeats could be fitted into the regions flanking the central region (Fig. 1*E* and *SI Appendix*, Fig. S6 *A* and *B*). Analogous to eEF3, the central eEF3-like region of Gcn1 contacts expansion segment 39 (ES39) and ribosomal proteins uS13 and eS19 in the head of the 40S subunit, as well as uL5 and uL18 in the central protuberance of the 60S subunit, of the leading ribosome (*SI Appendix*, Fig. S6 *C* and *D*). The flanking region N-terminal to the eEF3-like region of Gcn1 spans across the disome interface and establishes interactions with eS12 and eS31 within the beak of the 40S subunit of the colliding ribosome (*SI Appendix*, Fig. S6 *E* and *F*). Although we did not observe direct interactions between Gcn1 residues 1060 to 1777 and eS10, as suggested previously (17), we note that eS10 is adjacent to eS12 in the 40S head (*SI Appendix*, Fig. S6 *E* and *F*), and thus mutations or loss of eS10 could indirectly influence Gcn1 binding to the ribosome. While the central region of Gcn1 is relatively stable, the N- and C-terminal “arms” of Gcn1 are highly flexible and wind their way across the disome toward the factor binding site and the P-stalk of the colliding and leading ribosomes (Fig. 1 *B*–*E*). The N-terminal arm of Gcn1 contacts ES43L, uL11, and P0 at the stalk base, whereas the remaining 600 N-terminal amino acids fuse with density from the other P-stalk proteins, precluding any molecular interpretation (*SI Appendix*, Fig. S6 *E* and *F*). Similarly, the C-terminal arm of Gcn1 also reaches toward the factor binding site, but on the leading ribosome, where the C terminus appears to extend and contact the P-stalk proteins, although this interaction is also poorly resolved (*SI Appendix*, Fig. S6 *G* and *H*). The interaction between Gcn1 and the P-stalk seen here provides a likely explanation for the observation that mutations or loss of the P-stalk proteins impairs Gcn2-dependent eIF2 phosphorylation (18). We note that the P-stalk proteins have been shown in vitro to activate Gcn2-dependent eIF2 phosphorylation in the absence of Gcn1 (19).

The conformation of the leading and colliding ribosomes within the Gcn1-bound disome are distinct from one another. The leading ribosome is in a nonrotated pretranslocational state with a peptidyl-tRNA in the A-site, a deacylated tRNA in the P-site, and the elongation factor eIF5A in the E-site (Fig. 1*F*). The presence of eIF5A in the Gcn1-disome suggests that translation by the leading ribosome may have slowed, or even stalled, due to the presence of problematic polypeptide motifs at the peptidyl-transferase center of the large subunit (20, 21). By contrast, the colliding ribosome adopts a rotated hybrid state with a peptidyl tRNA in the hybrid A/P-site and a deacylated tRNA in the P/E-site (Fig. 1*G*). In this case, peptide bond formation has ensued, but translocation of the mRNA and tRNAs on the small subunit has not occurred. The overall constellation of a nonrotated leading ribosome followed by a rotated colliding ribosome observed in our Gcn1-disome is reminiscent of that observed previously for other colliding disomes, namely disomes formed in the presence of an inactive eRF1^AAG^ mutant (22) or stalled on CGA-CCG– and CGA-CGA–containing mRNAs (23, 24), but differs most greatly from the poly(A)-stalled disomes that also contained nonrotated colliding ribosomes (25) (*SI Appendix*, Fig. S7).

### Visualization of Rbg2-Gir2 on the Leading Ribosome of the Gcn1-Disome.

**Fig. 2.**
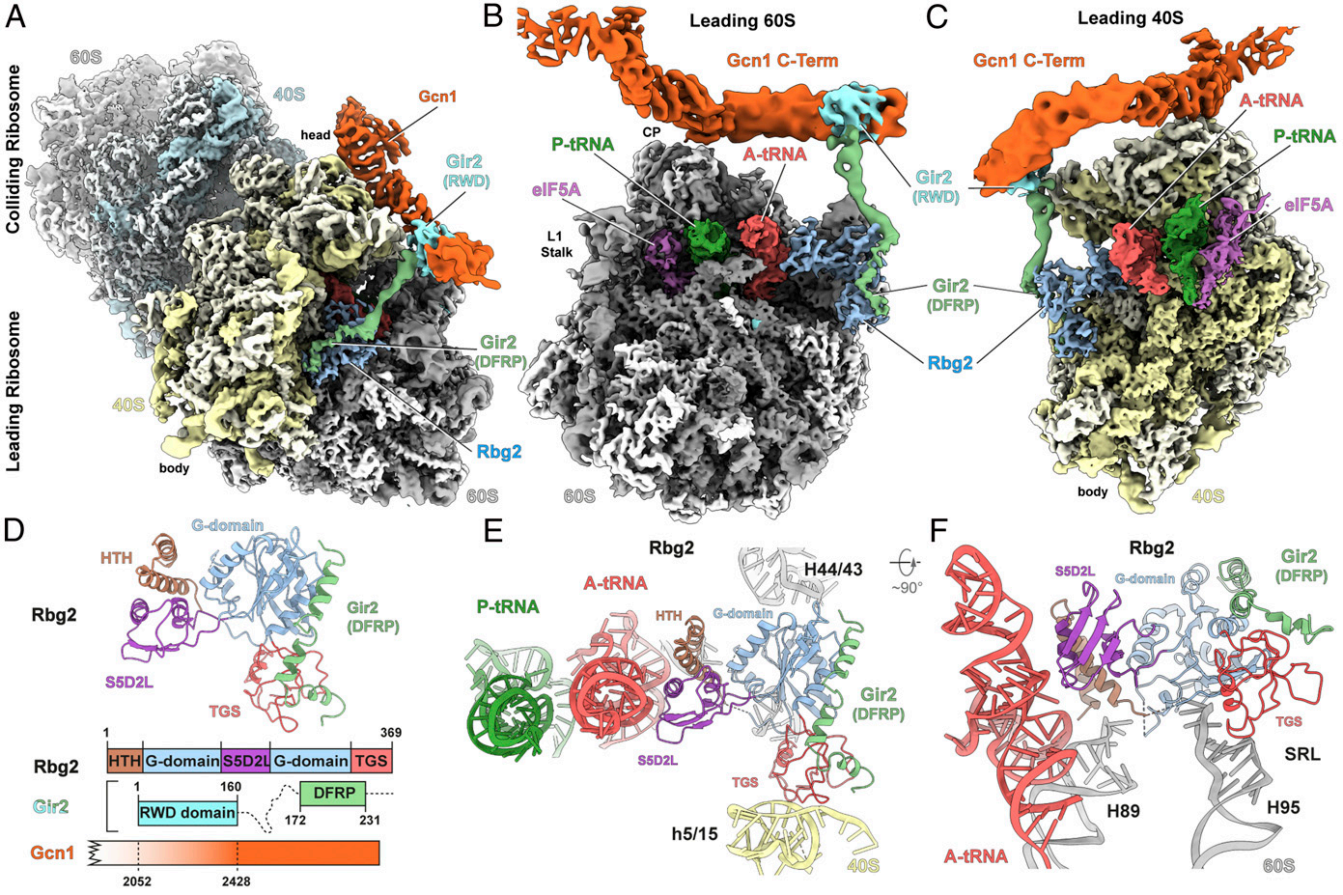
Structure of Rbg2-Gir2 on the leading stalled ribosome. (*A*–*C*) Cryo-EM reconstruction of the Gcn1-disome complex (*A*) and interface views of the 60S subunit (*B*) and 40S subunit (*C*) of the leading stalled ribosome. Segmented densities for Gcn1 (orange), colliding ribosome (40S, cyan; 60S, gray), the leading ribosome (40S, pale yellow; 60S, gray), P-tRNA (green), A-tRNA (red), Rbg2 (light blue), Gir2-DFRP (green), Gir2-RWD (cyan), and eIF5A (purple). (*D*) Molecular model for Rbg2-Gir2-DFRP with schematic representation of the Rbg2-Gir2-Gcn1 interactions. Domains are colored as indicated. (*E* and *F*) Interactions of Rbg2 colored by domain as in *D* with 40S (pale yellow) and 60S (gray) components and A-site tRNA (red) and P-site tRNA (green).

In addition to the presence of eIF5A in the E-site, the leading ribosome of the Gcn1-disome contained additional density within the factor binding site, adjacent to the A-site, which resembled a GTPase but not one of the canonical translational GTPases (Fig. 2 *A*–*C*). A mass spectrometry analysis of the Gcn1-disome sample instead revealed the presence of the noncanonical ribosome-binding GTPase 2 (Rbg2), which had comparable intensities to ribosomal proteins as well as some ribosome-associated factors, such as eIF5A and Mbf1 (Dataset S1). Although there is no available structure for Rbg2, it was possible to generate a homology model based on the structure of the closely related (62% identity) Rbg1 (26), which could then be satisfactorily fitted to the cryo-EM density (*SI Appendix*, Fig. S8 *A*–*C*). Like Rbg1, Rbg2 comprises four domains: an N-terminal helix-turn-helix (HTH), a C-terminal TGS (ThrRS, GTPase, and SpoT), and a central GTPase domain (G-domain) that is interrupted by a ribosomal protein S5 domain 2-like (S5D2L) domain (Fig. 2*D*). While the binding site of Rbg2 overlaps that of other GTPases, such as eEF2 and Hbs1, the overall architecture is distinct (*SI Appendix*, Fig. S8 *D*–*F*).

In the Gcn1-disome, the TGS domain of Rbg2 interacts with the 40S subunit, contacting helix 5/15 (h5/15) of the 18S rRNA, whereas the G and HTH domains establish contacts with the 60S subunit, including the stalk base (H43/H44), sarcin-ricin loop (SRL; H95), and H89 (Fig. 2 *E* and *F*). By contrast, the S5D2L domain of Rbg2 makes contacts exclusively with the A-site tRNA, such that α-helix α7 of Rbg2 approaches the minor groove of anticodon stem in the vicinity of nucleotides 27 to 29 of the A-site tRNA (Fig. 2 *E* and *F*). This suggests that Rbg2 can stabilize the accommodated A-site tRNA and thus may work together with eIF5A to facilitate peptide bond formation at problematic peptide motifs. Rbg1 and Rbg2 homologs are encoded in the majority of eukaryotes, with the mammalian counterparts termed developmentally regulated GTP-binding proteins 1 (DRG1) and DRG2, respectively (27). The very high conservation (66% identity) between human (DRG1/2) and yeast (Rbg1/2) orthologs suggests that the interactions observed here for Rbg2 are likely identical for DRG2 on the human ribosome.

Under physiological conditions, Rbg2 is very labile but becomes stabilized through interactions with Gir2, whereas in contrast, Rbg1 forms a complex with Tma46 (13). Both Gir2 and Tma46 contain a C-terminal DRG family regulatory protein (DFRP) domain that is critical for interaction with Rbg2 and Rbg1, respectively (12, 13, 28). In the Rbg1-Tma46(DFRP) X-ray structure, four α-helices at the C terminus of the DFRP domain of Tma46 establish contact with the TGS and G domain of Rbg1 (26) (*SI Appendix*, Fig. S8*G*). Consistently, we observe an analogous interaction between the DFRP domain of Gir2 and the TGS and G domains of Rbg2 (Fig. 2*D* and *SI Appendix*, Fig. S8*H*); however, unlike Tma46, where the linker region wraps around the G domain of Rbg1, the linker region of Gir2 extends away from Rbg2 toward Gcn1 (Fig. 2 *A*–*C* and *SI Appendix*, Fig. S8 *H* and *I*). This suggests that in the absence of the ribosome, the intimate interaction between Tma46/Gir2 and Rbg1/Rbg2 stabilizes their respective complexes, whereas upon ribosome binding, the N-terminal domains are freed to find new interaction partners. With respect to Rbg2-Gir2 in the Gcn1-disome structure, we observe that the density for the N-terminal region of Gir2 fuses with the C-terminal region (residues 2000 to 2200) of Gcn1 (Fig. 2 *A*–*C*).

Although the contact cannot be resolved in any detail owing to the high flexibility within this region, support for such an interaction is well documented. The N-terminal RWD domain of Gir2 is necessary and sufficient for interaction with Gcn1, and a construct containing only the C-terminal residues 2048 to 2382 of Gcn1 retains the ability to bind Gir2 (11). Moreover, the ribosome association of Gir2 also has been shown to be partially dependent on the presence of Gcn1 (11). Thus, taken together with the biochemical studies, our structural findings reveal that Gir2 indeed acts as a physical link between Rbg2 and Gcn1 and might prevent Gcn2 activation in situations where Rbg2 mediates successful restoration of translation of the leading ribosome by stimulating peptide bond formation. However, we note that a *GIR2* deletion did not alter the level of *GCN4* expression, suggesting that Gir2 likely does not have a general role in controlling Gcn2 activity (11).

### Visualization of Mbf1 on the Colliding Ribosome of the Gcn1-Disome.

**Fig. 3.**
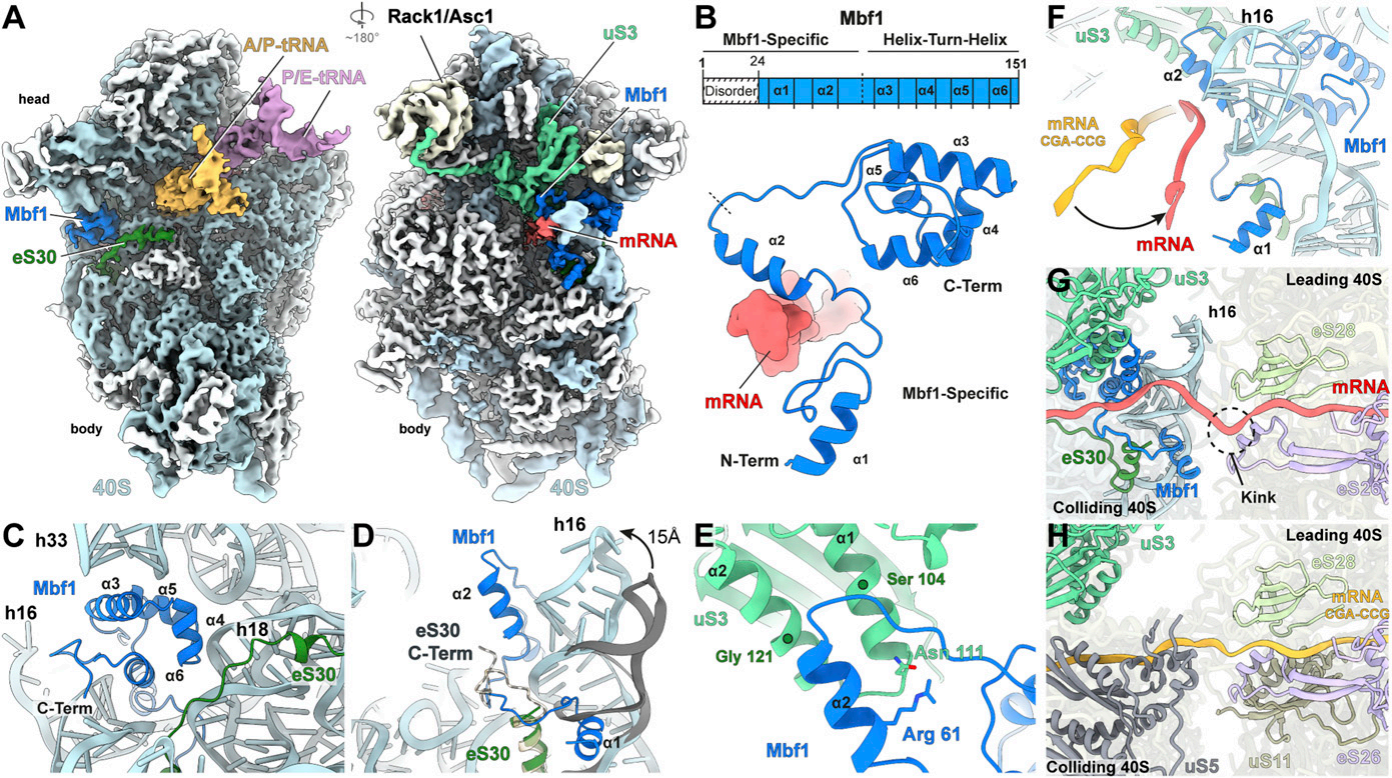
Structure of Mbf1 on the colliding ribosome. (*A*) Cryo-EM map of the interface (*Left*) and solvent (*Right*) view of the 40S subunit from the colliding ribosome, with 40S proteins (white), 18S rRNA (cyan), A/P-tRNA (gold), P/E-tRNA (purple), eS30 (dark green), uS3 (light green), mRNA (red) and Mbf1 (deep blue). (*B*) Schematic and cartoon representation of the Mbf1 molecular model (deep blue). (*C*) Mbf1 helix-turn-helix motif bound to the 40S subunit. (*D*) Refolding of h16 and eS30 C terminus destabilization on Mbf1 binding. Comparison of h16 (cyan) and eS30 (dark green) in the Mbf1-bound colliding ribosome to h16 (gray) and eS30 (light gold) in the colliding ribosome of an Mbf1-lacking disome (Protein Data Bank [PDB] ID 6SNT) (24) (*E*) Close-up view of uS3 and Mbf1 helix 2 interactions. (*F*) Comparison of the path of the mRNA (red) in the Mbf1-bound structure compared to the mRNA (orange) in a colliding ribosome of an Mbf1-lacking disome (PDB ID 6I7O) (23). (*G* and *H*) The mRNA path between the 40S of the colliding ribosome and the 40S of the leading ribosome within the Gcn1-disome (G) and the CGA-CCG stalled disome (PDB ID 6I7O) (*H*) (23). Components interacting with the mRNA at the 40S-40S interface are shown for each disome, respectively.

Within the colliding ribosome of the Gcn1-disome, we observed additional density located between the head and body of the 40S subunit that we attributed to Mbf1 (Fig. 3 *A* and *B* and *SI Appendix*, Fig. S9*A*), a conserved archaeal/eukaryotic protein that suppresses +1 frameshifting at inhibitory CGA-CGA codon pairs in yeast (29). Recent findings indicate that the mammalian Mbf1 homolog, EDF1, stabilizes GIGYF2 at collisions to inhibit translation initiation in cis (30, 31); however, in our reconstruction, we did not observe any additional density for the yeast GIGYF2 homologs (Smy2/Syh1) or detect them in our mass spectrometry data (Dataset S1). The assignment of Mbf1 was based on the high intensity of Mbf1 peptides in the mass spectrometry analysis of the Gcn1-disome sample (Dataset S1) and the excellent agreement between the cryo-EM density and a homology model for Mbf1 generated from the NMR structure of the C-terminal HTH domain of Mbf1 from the fungus *Trichoderma reesei* (32) (*SI Appendix*, Fig. S9 *B* and *C*). Moreover, the binding site for Mbf1 observed here on the Gcn1-disome is consistent with that observed recently on stalled disomes/trisomes (31). The C-terminal HTH domain of Mbf1 connects h33 in the head with h16 and h18 within the body of the 40S subunit (Fig. 3*C*), thereby stabilizing a nonswiveled conformation of the head (*SI Appendix*, Fig. S9 *D*–*F*). Interaction with h33 is likely critical for Mbf1 function, since mutations (I85T, S86P, R89G, and R89K) within helix α3, which contacts h33, leads to loss of frameshift suppression (29). Binding of Mbf1 leads to a shift of h16 toward the body of the 40S subunit (Fig. 3*D*), which is stabilized by interactions of the C terminus and helix α6 of Mbf1 with the minor groove of h16 (Fig. 3*D*). In addition to the HTH domain, we were able to model the N-terminal residues 25 to 79 of Mbf1, including two short α-helices, α1 and α2, formed by residues 26 to 37 and 59 to 68 (Fig. 3*B*). Helix α2 of Mbf1 interacts directly with helices α1 and α2 of ribosomal protein uS3 (Fig. 3*E*). These interactions are likely essential for Mbf1 function, since S104Y and G121D substitutions within these two helices of uS3 result in a loss of frameshift suppression, which can be partially restored by overexpression of Mbf1 (29). Mutations (R61T and K64E) located in the distal region of helix α2 of Mbf1 also abolish frameshift suppression (29). Arg61 comes into hydrogen-bonding distance with Asn111 of S3 (Fig. 3*E*), and Lys64 appears to interact with the backbone phosphate oxygens of h16. Asc1 (RACK1) is also critical for suppressing +1 frameshifting at CGA repeats (29, 33); however, our structure suggests that this is not due to direct interaction with Mbf1, but rather because Asc1 appears to be critical for disome formation by establishing multiple interface contacts between the leading and colliding ribosomes (*SI Appendix*, Fig. S7).

The N-terminal residues preceding helix α2 of Mbf1 wrap around the stem of h16, and consequently, the C terminus of eS30e becomes disordered (Fig. 3 *D* and *F* and *SI Appendix*, Fig. S9 *G*–*I*). Helix α1 of Mbf1 is located within the major groove of h16 oriented toward the interface, suggesting that N-terminal 24 amino acids that are not observed in our structure may reach toward the leading ribosome (Fig. 3 *F* and *G*). The shift of h16 induced by Mbf1 brings the minor groove of h16 into contact with the mRNA, which, together with the direct interaction observed between the mRNA and helix α2 of Mbf1, causes a redirection in the path of the 3′ end of the mRNA compared to the CGA-CGG stalled disome structure (Fig. 3*F*) (23, 24). Moreover, the mRNA appears to be kinked at the interface between the leading and colliding ribosomes, suggesting the adoption of a relaxed state instead of the more extended and potentially strained conformation seen in other disomes (Fig. 3*G*). Ribosome collisions have been shown to induce +1 frameshifting because the colliding ribosome exerts a pulling force on the mRNA during translocation that promotes slippage of the mRNA with respect to the tRNAs in the leading ribosome (34). Our findings suggest that Mbf1 suppresses +1 frameshifting on the leading ribosome by binding to the colliding ribosome and locking the 40S subunit in such a manner that mRNA movement is prevented. Specifically, Mbf1 prevents the head swiveling that is required for mRNA/tRNA translocation and stabilizes the mRNA via direct interactions, as well as indirectly by promoting additional interactions between the mRNA and the ribosome, especially h16. Finally, we note that the overall arrangement of the leading and colliding ribosomes in the Gcn1-disome is more compact than those observed for the CGA-CCG disome (23, 24) (Fig. 3 *G* and *H* and *SI Appendix*, Fig. S10 *A*–*F*). This results in a shorter path that the mRNA needs to traverse between the ribosomes, which may also contribute to maintaining a relaxed mRNA conformation on the leading ribosome. These findings imply that Gcn1 interaction with the 40S subunit of the colliding ribosome (*SI Appendix*, Fig. S6 *E* and *F*), rather than Mbf1–40S interactions, are likely critical for promoting the novel compact architecture of the Gcn1-disome. Furthermore, because Mbf1 makes no contacts with Gcn1 or Rbg2, we have no reason to believe that the latter are instrumental in recruiting Mbf1 to a disome or in supporting the suppression of frameshifting at problematic codons by Mbf1.

## Discussion

**Fig. 4.**
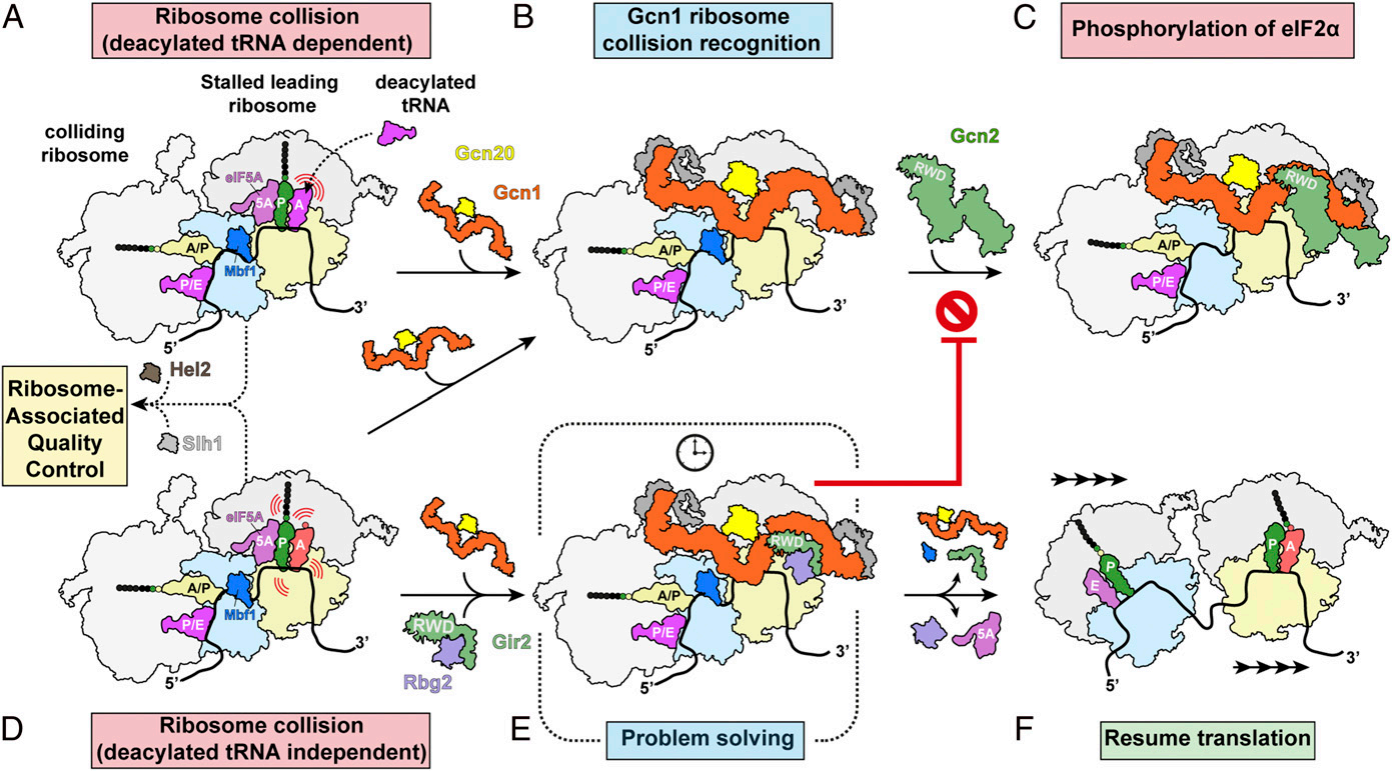
Gcn1 as a checkpoint for disome collision. (*A*) Amino acid starvation leads to increased binding of uncharged tRNAs within the ribosomal A-site, leading to translation slowdown/stalling and collisions. (*B*) Colliding ribosomes (disomes) are recognized by Gcn1-Gcn20. (*C*) Gcn1 in turn recruits Gcn2 via direct interaction with the Gcn2 N-terminal RWD domain. Activation of Gcn2 results in the phosphorylation of eIF2α and induction of the GAAC pathway. (*D*) Translating ribosomes may also encounter specific mRNA sequences/structures and/or nascent polypeptide motifs that induce a translational slow-down or pausing, also leading to collisions and disome formation, which are known as substrates for the ribosome quality control but are also recognized by Gcn1. (*E*) Concomitant recruitment of the Rbg2-Gir2 to the leading ribosome by Gcn1 allows Rbg2 to resolve the slowdown, while Gir2 prevents the recruitment and activation of Gcn2 and eventually allows translation to resume (*F*).

Together with the available literature, our findings lead us to present a model for how Gcn1 could sense stalled ribosomes and subsequently recruit Gcn2 to the stalled disome. Under conditions of amino acid starvation, the binding of deacylated tRNA in the A-site of the ribosome causes translational stalling, which in turn increases the frequency of ribosome collisions and disome formation (35, 36) (Fig. 4*A*). We envisage that such disomes are recognized by the Gcn1-Gcn20 complex in an analogous manner to that observed here (Fig. 4*B*), and that Gcn1 can recruit and activate Gcn2 via direct interaction with its N-terminal RWD domain (Fig. 4*C*), analogous to the interaction established between Gcn1 and the RWD domain of Gir2. Moreover, there is growing evidence that Gcn2 activation also occurs in response to stimuli that promote collisions, but in a deacylated tRNA-independent manner (37–40) (Fig. 4*D*). This is consistent with our structural findings suggesting that Gcn1 recognizes the architecture of a disome, rather than directly monitoring the presence or absence of an A-site tRNA. Thus, an important prediction that we make from our study is that recognition of colliding ribosomes or disomes enables Gcn1 to facilitate Gcn2 activation in response to many diverse environmental stresses that act to inhibit translation.

Furthermore, the discovery of Rbg2-Gir2 bound to the leading ribosome of the Gcn1-disome provides support for the previously proposed concept that Gcn1 acts as a scaffold to interact with other accessory factors and thereby fine-tune the level of Gcn2 activation (2). One such example of this is the Rbg2-Gir2 complex, which is known to repress Gcn2 activation upon overexpression (11). Recent findings suggest that Rbg2 (and Rbg1) facilitate translation through problematic polybasic (Arg/Lys-rich) stretches in proteins (41). We observe Rbg2 interacting with the A-site tRNA on the leading ribosome of the Gcn1-disome, suggesting that it may stabilize the A-tRNA to promote peptide bond formation and restore the translational activity of the stalled ribosome (Fig. 4 *E* and *F*). Moreover, we suggest that during this “problem-solving” phase, Gcn2 recruitment and activation are prevented owing to the competing interaction of the RWD domain of Gir2 with Gcn1 (11) (Fig. 4*E*). However, as noted above, deletion of *GIR2* did not alter the level of GCN4 expression, suggesting that Gir2 likely does not play a general role in controlling Gcn2 activity (11). Wout et al. (11) suggested that Gir2 may inhibit Gcn2 activation under certain conditions or within specific subcellular locations when or where Gcn2 activation is unfavorable for the cell.

Finally, our finding that colliding ribosomes are the substrate for Gcn1 recruitment provides a rationale for the emerging link between Gcn2 activation and the ribosome quality control (RQC) pathway. The RQC pathway targets stalled ribosomes for disassembly and promotes degradation of aberrant mRNAs and nascent polypeptide chains (42–44). A central player in the RQC pathway is Hel2/ZNF598, which recognizes colliding ribosomes and ubiquitylates specific 40S ribosomal proteins. This in turn recruits the helicase Slh1/ASCC to dissociate the leading ribosome from the mRNA, allowing the following ribosomes to continue translating (24, 45). Deletion of Hel2 in yeast has been recently reported to cause an increase in eIF2 phosphorylation (36), and activation of RQC by Hel2 has been shown to suppress that of Gcn2 (46). In light of our results, a likely explanation for this observation is that in the absence of Hel2, additional disome substrates become available for Gcn1 binding, leading to increased Gcn2 activation and eIF2 phosphorylation. Like Hel2, Slh1 is also nonessential in yeast, but loss of Slh1 is synthetic lethal when combined with a Rbg1/Tma46/Rbg2/Gir2 quadruple knockout (26). This implies that for survival, eukaryotic cells must remove the translational roadblock through either reactivation of translation of the leading ribosome by, for example, Rbg2-Gir2 (Fig. 4 *E* and *F*), or disassembly of the stalled disome roadblock on the mRNA via the RQC pathway. While further work is needed to dissect out the mechanistic details and interplay between the factors and pathways, their conservation across all eukaryotes, including humans, implies a central and evolutionary importance.

## Materials and Methods

The TAP-Tag in vivo pull-out was performed using the GCN20 TAP-tagged strain (SC0000; MATa; ura3-52; leu2-3,112; YFR009w::TAP-KlURA3) Euroscarf, essentially as described previously (47). Cells were harvested at the mid log phase at an OD_600_ of 2.5 and lysed via glass bead disruption. The cleared lysate was incubated with IgG-coated magnetic Dynabeads M-270 epoxy (Invitrogen) for 1 h at 4 °C with slow tilt rotation. The elution was performed by addition of AcTEV Protease (Invitrogen) for 2 h at 17 °C in elution buffer containing 20 mM Hepes (pH 7.4). 100 mM KOAc, 10 mM Mg(OAc)_2_, 1 mM DTT, and 1 mM ADPNP (Sigma-Aldrich). Since Gcn20 is an ABC ATPase, and ATP is known to stabilize binding and thus enhance Gcn2 activation (4, 5, 7, 8, 48), we included the nonhydrolyzable ATP analog ADPNP in the last step of the purification. The pull-out sample was analyzed by 4% to 12% sodium dodecyl sulfate polyacrylamide gel electrophoresis and immunoblot analysis, as well as by mass spectrometry. Here 0.02% glutaraldehyde was added to the freshly eluted TAP-Tag pull-out complex, followed by incubation for 20 min on ice. The cross-linking reaction was quenched by the addition of 25 mM Tris⋅HCl (pH 7.5), and n-dodecyl-D-maltoside (DDM) was added to a final concentration of 0.01% (vol/vol). Then 5 µL (8 A_260_/mL) of the freshly purified and cross-linked complex was applied to 2-nm precoated Quantifoil R3/3 holey carbon supported grids and vitrified using a Vitrobot Mark IV (FEI). Low-resolution cryo-EM was performed on a 120-kV Tecnai G2 Spirit transmission electron microscope (FEI) equipped with a TemCam-F816 camera (TVIPS), whereas high-resolution cryo-EM was performed on an FEI Titan Krios transmission electron microscope operating at 300 kV equipped with a Falcon II direct electron detector. Automated particle picking was then performed using Gautomatch (https://www.mrc-lmb.cam.ac.uk/kzhang/) and processed using the RELION-3.0 software package (49). Molecular models were generated using SWISS-MODEL (50), UCSF Chimera 1.13.1 (51), Coot (52), and ISOLDE (53), and refinement was performed using PHENIX (54). Figures showing atomic models and electron densities were generated using either UCSF Chimera (51) or ChimeraX (55) and assembled with Inkscape (https://inkscape.org/) and Adobe Illustrator.

## Supplementary Material

Supplementary File

Supplementary File

Supplementary File

## Data Availability

The cryo-EM maps and associated molecular models for the leading stalled and colliding ribosomes have been deposited in the Electron Microscopy Data Bank (entries EMD-12534 and EMD-12535) and the Protein Data Bank (PDB ID 7NRC and 7NRD, respectively). Mass spectrometry data have been deposited in the ProteomeXchange database (dataset PXD021365).
