# Supplementary material for: Structure of Gcn1 bound to stalled and colliding 80S ribosomes
Source: Proc Natl Acad Sci U S A. 2021 Mar 31;118(14):e2022756118. doi: 10.1073/pnas.2022756118 (PMC8040806; doi:10.1073/pnas.2022756118)
Supplement: Supplementary File [file pnas.2022756118.sapp.pdf]

PNAS
www.pnas.org

Supplementary Information for
Structure of Gcn1 bound to stalled and colliding 80S ribosomes

Agnieszka A. Pochopien[1,4], Bertrand Beckert[1,4], Sergo Kasvandik[2], Otto Berninghausen[3], Roland Beckmann[3], Tanel Tenson[2] and Daniel N. Wilson[1,*]

[1] Institute for Biochemistry and Molecular Biology, University of Hamburg, Martin-Luther-King-Pl. 6, 20146 Hamburg, Germany.
[2] University of Tartu, Institute of Technology, 50411 Tartu, Estonia
[3] Gene Center and Department of Biochemistry, University of Munich, 81377 Munich, Germany
[4] These authors contributed equally.

*Daniel N. Wilson.
Email: Daniel.Wilson@chemie.uni-hamburg.de

This PDF file includes:

Supplementary text
Figures S1 to S10
Tables S1
Legends for Movie S1
Legends for Dataset S1
SI References

Other supplementary materials for this manuscript include the following:

Movie S1
Datasets S1

Supplementary Information Text

Preparation of cell extracts and sucrose gradient analysis

Yeast whole-cell extracts of the TAP-tagged GCN20 strain (SC0000; MATa; ura3-52; leu2-3,112; YFR009w::TAP-KlURA3; Euroscarf) were prepared from cultures grown to OD_{600} of 0.8-1.0 in either YPD or SC medium (plus supplements as required). Starvation conditions were induced by addition of 15 mM 3 amino-1,2,4-triazole (3-AT) (Sigma-Aldrich) to the SC culture 15 min before harvesting. Cells were harvested by centrifugation at 4,400 x g for 10 min at 4°C in a Sorvall SLC-6000 rotor (Marshall Scientific). The cell pellets were washed with ice cold water and lysis buffer (20 mM HEPES (pH 7.4), 100 mM KOAc, 10 mM $Mg(OAc)_2$, 1 mM DTT, Complete EDTA-free Protease Inhibitor cocktail (Roche)), transferred to a 50 ml falcon, resuspended in lysis buffer and disrupted using glass beads. Glass beads were removed by centrifugation for 5 min at 4,400 x g at 4°C in the Rotanta 460R falcon centrifuge (Hettich). The cell debris were further pelleted by centrifugation at 17,600 x g for 15 min at 4°C in a Sorvall RC 6 SS-34 rotor. The cleared lysate was layered onto 12 ml 10–60% sucrose gradient prepared in lysis buffer and centrifuged in an SW-41Ti rotor (Beckman Coulter) for 5 h at 99,000 x g at 4°C. The ribosomal fractions were analysed using a Gradient Station (Biocomp) with an Econo UV Monitor (Bio-Rad) (Fig. S1A).

Tandem affinity purification for cryo-EM analysis and Immunoblotting

The native TAP-Tag pull-out was performed using the GCN20 (and GCN1) TAP-tagged strain essentially as described before (1). Yeast whole-cell extracts of TAP-tagged GCN20 strain (SC0000; MATa; ura3-52; leu2-3,112; YFR009w::TAP-KlURA3; Euroscarf), were prepared from cultures grown in YPD or SC media (plus supplements as required). Cells grown in YPD media were harvested at the mid log phase at an OD_{600} of 2.5. Cells cultured in the SC media were grown until OD_{600} of 0.8 and starvation was induced for 15 min by addition of 15 mM 3-AT (Sigma-Aldrich) before harvesting. Both cell pellets were lysed via glass bead disruption and the cleared lysates were incubated with IgG-coated magnetic DynabeadsR©M-270 Epoxy (Invitrogen) for 1 h at 4°C with slow tilt rotation. The elution was performed by addition of AcTEV Protease (Invitrogen) for 2 h at 17°C in elution buffer containing 20 mM HEPES (pH 7.4). 100 mM KOAc, 10 mM $Mg(OAc)_2$, 1 mM DTT and 1 mM ADPNP (Sigma-Aldrich). The pull-out samples were analyzed by 4-12% NuPAGE gel (Thermofisher) and immunoblot analysis (Fig. S1B,C). For the latter one, the membrane with the transferred protein bands was shortly washed with phosphate-buffered saline (PBS) (pH 7.4) and blocked 3 x 15 min with 2% milk/PBS at RT. The blocked membrane was incubated for 1 h with the first TAP-tag polyclonal antibody (CAB1001, Rabbit/IgG, Thermofisher #CAB1001), washed 3x15 min with PBS and incubated with the second anti-rabbit HRP antibody (Goat/IgG, Thermofisher #31460) ON at 4°C. The next day, the membrane was shortly washed with PBS and protein bands were detected using Pierce^{TM} ECL Western Blotting Substrate (Thermofisher #32109) and the Amersham Imager600 (GE Healthcare). Control purifications were

also performed using an untagged wildtype strain, where no purification of ribosomal proteins was observed, as reported previously (2), suggesting that the ribosomal complexes observed in the TAP-tagged strains co-purified with the Gcn20. Moreover, purifications performed using eEF3-TAP strains did not enrich for Gcn1, Rbg2, Gir2 or Mbf1 (2), as we observed here for Gcn20-TAP, suggesting that the detection of these proteins is specific for the Gcn20 pull-out complex.

Detection of eIF2 phosphorylation

The yeast cell extract of the GCN20-TAP strain was prepared from culture grown to log-phase (OD_{600} of 0.8-1.0) in SC medium and starvation was induced by addition of 1, 5 or 15 mM 3-AT for 15 min before harvesting. 1 ml of cells with an OD_{600} of 1 was transferred into a 1.5 ml Eppendorf tube and centrifuged in a 5417R Microcentrifuge (Eppendorf) for 5 min at 20,000 x g at 4°C. The cell pellet was resuspended in 1 ml of ice-cold water and 150 µL of NaOH/β-Me buffer (2 M NaOH and 1.4 M β-Mercaptoethanol) was added to the sample and incubated on ice for 15 min with occasional vortexing. Subsequently, 150 µL 72% (w/v) TCA was added to each sample and further incubated on ice for 15 min and then centrifuged at 4°C, 25,000 x g for 20 min. After removal of the supernatant, the dried pellets were resuspended in 50 µL HU buffer (200 mM PBS (pH 7.4), 8 mM Urea, 5% (w/v) SDS, 1 mM EDTA, 100 mM freshly added DTT), denatured by heating 15 min at 65°C, separated on 10% SDS polyacrylamide gel and subjected to immunoblot analysis as described before. Phosphorylation level of eIF2α was detected using eIF2α antibody (Rabbit, Cell Signalling #3597) while PGK1, which was used as loading control, was detected using the commercially available PGK1 antibody (Mouse, Abacam #113687) (**Fig. S1D**).

Proteomics sample preparation and nano-LC/MS/MS analysis

Gcn20-pullout sample was loaded onto SDS-PAGE gels and run for a short time so that the samples entered into the gel and then the complete sample was excised from the gel as a single band. The gel band was then destained in 1:1 acetonitrile (ACN):100 mM ammonium bicarbonate (ABC) with vortexing, reduced with 10 mM dithiothreitol at 56ºC and alkylated with 50 mM chloroacetamide in the dark. Protein digestion was carried out overnight with 10 ng/µl of dimethylated porcine trypsin (Sigma Aldrich) in 100 mM ABC at 37ºC. Peptides were extracted from the gel matrix using bath sonication, followed by 30 min vortexing in 2 volumes of 1:2 5% formic acid (FA): ACN. The organic phase was evaporated in a vacuum-centrifuge, after which the peptides were desalted on in-house made C18 (3M) solid phase extraction tips. Purified peptides were reconstituted in 0.5% trifluoroacetic acid. Peptides were injected to an Ultimate 3000 RSLCnano system (Dionex) using a 0.3 × 5 mm trap-column (5 µm C18 particles, Dionex) and an in-house packed (3 µm C18 particles, Dr Maisch) analytical 50 cm × 75 µm emitter-column (New Objective). Peptides were eluted at 200 nL/min with an 8-40% (30 min) A to B gradient (buffer A: 0.1% (v/v) FA; buffer B: 80% (v/v) ACN + 0.1% (v/v) FA) to a quadrupole-orbitrap Q Exactive Plus

(Thermo Fisher Scientific) MS/MS via a nano-electrospray source (positive mode, spray voltage of 2.5 kV). The MS was operated with a top-10 data-dependent acquisition strategy. Briefly, one 350-1,400 m/z MS scan at a resolution setting of R = 70,000 was followed by higher-energy collisional dissociation fragmentation (normalized collision energy of 26) of the 10 most intense ions (z: +2 to +6) at R = 17,500. MS and MS/MS ion target values were 3,000,000 and 50,000 ions with 50 and 100 ms injection times, respectively. Dynamic exclusion was limited to 15 s. MS raw files were processed with the MaxQuant software package (version 1.6.1.0) (3). Methionine oxidation, protein N-terminal acetylation were set as potential variable modifications, while cysteine carbamidomethylation was defined as a fixed modification. Identification was performed against the UniProt (www.uniprot.org) *Saccharomyces cerevisiae* (strain ATCC 204508 / S288c) reference proteome database using the tryptic digestion rule. Only identifications with at least 1 peptide ≥ 7 amino acids long (with up to 2 missed cleavages) were accepted. Intensity-based absolute quantification (iBAQ) (4) feature of MaxQuant was enabled. This normalizes protein intensities by the number of theoretically observable peptides and enables rough intra-sample estimation of protein abundance. Peptide-spectrum match, peptide and protein false discovery rate was kept below 1% using a target-decoy approach (5). All other parameters were default. Data are available via ProteomeXchange with identifier PXD021365.

Sample and grid preparation

0.02% glutaraldehyde were added to the freshly eluted TAP-Tag pull-out complex and incubated for 20 min on ice. The crosslinking reaction was quenched by addition of 25 mM Tris-HCl (pH 7.5) and n-dodecyl-D-maltoside (DDM) was added to a final concentration of 0.01% (v/v). 5 µL (8 A_{260}/mL) of the freshly purified and crosslinked complex was applied to 2 nm precoated Quantifoil R3/3 holey carbon supported grids and vitrified using a Vitrobot Mark IV (FEI, Netherlands).

Low resolution data collection and image processing

The TAP-Tag *in vivo* Gcn20 pull-out sample was initially checked by generating a low resolution cryo-EM reconstruction with a dataset consisting of 264 micrographs collected on a 120 kV Tecnai G2 Spirit (FEI) transmission electron microscope (TEM) equipped with a TemCam-F816 camera (TVIPS) at a pixel size of 2.55 Å with a defocus range of −3.5 to −1.5 µm. Particle picking was performed automatically using Gautomatch (http://www.mrc-lmb.cam.ac.uk/kzhang/) resulting in 40,012 particles (**Fig. S2**). An initial 3D reconstruction was obtained using a vacant *S. cerevisiae* 80S ribosome as a reference, followed by 3D classification into 5 classes. When compared to the vacant 80S ribosome, class 1 (5%; 2,033 particles) displayed additional density within the A-site factor binding site as well as a tube-like extra density spanning the head and central protuberance of the 80S ribosome. In addition, the class 1 ribosome displayed clear density for a neighbouring ribosome, suggesting the presence of a disome. Class 2 (14%; 5,589 particles) revealed a vacant

80S ribosome whereas class 3 (17%; 6,809 particles) had some density within the A-site region. Finally, class 4 and 5 were attributed to ´junk´ classes. The particles from class 1 were selected and re-extracted with bigger box size (increased from 420 to 700) in order to encompass the full neighbouring ribosome and subsequently 3D refined at around 21 Å resolution, revealing the structure of disome with the extra ´worm-like´ density for Gcn1 spanning both ribosomes within the disome (**Fig. S2**).

High resolution cryo-EM data collection and image processing

To obtain high resolution of the Gcn1-disome complex, a total of 16,823 micrographs with a total dose of 25 $e^{-}/Å^{2}$ at a nominal pixel size of 1.084 Å and with a defocus ranging from −2.8 to −1.3 μm were collected using the EPU software (Thermo Fisher) on a FEI Titan Krios TEM (Thermo Fisher) operating at 300 kV equipped with a Falcon II direct electron detector. Each micrograph, consisting of a series of 10 frames, was summed and corrected for drift and beam-induced motion using MotionCor2 (6). The power spectra, defocus values, astigmatism and estimation of micrograph resolution were determined using Gctf (7). Automated particle picking was then performed using Gautomatch (http://www.mrc-lmb.cam.ac.uk/kzhang/), yielding initially 949,522 particles that were subjected to 2D classification using the RELION-3.0 software package (8) (**Fig. S3**). After 2D classification, 616,079 particles were selected and subsequently subjected to 3D refinement using a vacant *S. cerevisiae* 80S ribosome as initial reference. The initially refined particles were further 3D classified into 15 classes (**Fig. S3**). Class 1 (8.6%, 52,933 particles) was identified as the leading stalled ribosome containing densities for A- and P-site tRNAs, eIF5A, Gcn1, Rbg2 and Gir2, whereas class 2 (6.5%, 39,575 particles) was identified as the collided ribosome with densities for Gcn1, Mbf1, A/P- and P/E-site tRNAs. Class 3 (24.2%, 149,887 particles) and class 4 (24.4%, 151,167 particles) represent the major classes, both containing tRNAs. Class 3 had rotated ribosomes with hybrid A/P- and P/E-site tRNAs, analogous to that observed previously (9, 10), whereas class 4 was non-rotated with A- and P-site tRNAs. In addition, class 4 had additional density in the A-site that would be consistent with an open conformation of eEF1A that occurs after tRNA release and GTP hydrolysis (11). Class 5 (10.6%, 65,173 particles) contained non-rotated ribosomes with P- and E-site tRNAs and additional density for eRF1 in the A-site and eEF3 bound to the head and central protuberance of the 40S and 60S, respectively. Further sub-sorting of this class revealed subpopulations containing eEF3 and eRF1, eRF1 and eIF5A, as well as eRF1, substoichiometric eRF3 and eIF5A, however, these subpopulations could not be refined to high resolution due to the low particle number. Class 6 (7.7%, 47,171 particles) contained hibernating 80S ribosomes with the presence of eEF2 and Stm1, as reported previously (12, 13). Class 7 (1.3%, 8,169 particles) contained mature 60S particles, whereas the remaining classes (classes 8-15 totalling 16.7%, 102,004 particles) contained damaged and/or non-aligning particles are were considered as low resolution/junk.

In order to ensure that Gcn1-containing particles were not lost during the initial 3D classification, all classes (1-15) were pooled together and sub-sorted again but using a mask to focus sorting on Gcn1. This resulting in a major class with 88,453 particles. Sub-sorting of this class produced a high-resolution class (class 1 with 30,016 particles) as well as two low resolution or orientation biased classes (classes 2 and 3, totally 58,437 particles). The particles from class 1 were selected and re-extracted with bigger box size (increased from 420 to 700) in order to encompass the full neighbouring ribosome. Following 3D refinement, CTF refinement yielded a structure of the full disome (**Fig. S3**) with an average resolution of 4.0 Å for the leading stalled and 8.4 Å for the colliding ribosome (**Fig. S4A-C**). Local refinement of the of the individual ribosomes yielded average resolutions of 3.9 Å (**Fig. S4D-G**) and 4.4 Å, respectively (**Fig. S4H-K**). Local refinement of aligned particles was also performed on the 40S head/Gcn1 region of the leading stalled ribosome (**Fig. S3**), which improved the local resolution of Gcn1 (**Fig. S4L-O**). Finally sharpening of the final maps was performed by dividing the maps by the modulation transfer function of the detector and by applying an automatically determined negative B factor to the maps using Relion-3.0. For model building the final maps were locally filtered and the local resolution estimated using Relion-3.0. The final resolution of each volume was determined using the “gold standard” criterion (FSC = 0.143).

Molecular Modelling

A homology model of the Gcn1 eEF3-like HEAT repeat region (predicted residue 1330-1641) was created using the 80S-bound eEF3 model (this study) as a template for SWISS-MODELLER (14). Comparison of the two cryo-EM maps of the ribosome bound by eEF3 and Gcn1 revealed an identical overall conformation and ribosomal binding site of eEF3-HEATs and Gcn1 eEF3-like HEATs. Based on that similarity, the created Gcn1 model (8 HEAT repeats; residues 1324-1638) was fitted (cross-correlation (CC) of 0.75 between model and map) into the appropriate 7 Å low-pass filtered cryo-EM map using the command ´fit in map´ in UCSF Chimera 1.13.1 (15) and manually adjusted with Coot version 0.8.9.2 (16). Beyond the Gcn1 EF3-like HEAT repeat region, three poly-alanine HEAT repeats were *de novo* modelled in the N-terminal direction (residues 1216-1323; (CC of 0.63 between model and map) and eight C-terminal HEAT repeats (residues 1639-1922; cross-correlation of 0.65 between model and map) using Coot version 0.8.9.2 (16). The peripheral N- and C-terminal regions could not be modelled due high flexibility of these regions in the cryo-EM map, however, the tube-like features of the density is consistent with secondary structure predictions of additional HEAT repeats, therefore, we tentatively fitted HEAT repeats from residues ~600-1100 (CC of 0.50 between model and map) and ~1950-2600 (CC of 0.57 between model and map). The very N- and C-terminal regions fuse with the stalk proteins and could not be modelled. Although Gcn20 was not well resolved, the location of the density and interaction region with Gcn1 would be consistent with the N-terminal region of Gcn20 based on available literature.

The extra density at the A-site entry factor of the stalled-leading ribosome was identified and modelled as Rbg2-Gir2. The crystal structure of the Rbg1 protein (PDB ID 4A9A) (17) was rigid body fitted into the isolated density using Chimera and the structure of Rgb2 was then generated by homology modelling within Coot (16) and Isolde (18) and subsequently refined in Phenix (19). Gir2-DFRP model was obtained using a homology model based on the Tma46 template from PDB ID 4A9A. However, due to the lack of side chain information, Gir2-DFRP has been modelled only as poly-Ala. Finally, to generate a full molecular model for the stalled leading Gcn1-Rbg2-Gir2-80S complex, existing models for the translating *S. cerevisiae* ribosome (PDB ID 6I7O), Phe-tRNA for A-tRNA and P-tRNA (PDB ID 1EVV), eIF5A (PDB ID 5GAK) (20) were used and combined with the Rbg2-Gir2 and Gcn1 model. The molecular model was then refined using Phenix (19).

The extra density located between the head and body of the colliding ribosome was identified and modelled as Mbf1 protein. The NMR structure of the C-terminal part of Mbf1 protein from the fungus *Trichoderma reesei* (PDB ID 2JVL) was rigid body fitted into the isolated density using Chimera and the *S. cerevisiae* structure of Mbf1 C-terminal region (residues 80-140) was then modelled by homology in Coot, while the N-terminal region (residues 24-79) was *de novo* modelled using Coot and Isolde. Helix 1 of Mbf1 was placed in the extra-density located in the major groove of h16, however, due to the lack of resolution, the model is only tentative and consists of a polyalanine trace. To generate a full molecular model for the colliding Gcn1-Mbf1-80S complex, existing models for the translating *S. cerevisiae* ribosome PDB ID 6I7O), Phe-tRNA (PDB ID 1EVV) were used and combined with the Mbf1 and Gcn1 model. The molecular model was then refined using Phenix.

Figure preparation

Figures showing atomic models and electron densities were generated using either UCSF Chimera (15) or Chimera X (21) and assembled with Inkscape (https://inkscape.org/) and Adobe Illustrator.

Data Availability

The cryo-EM maps of the leading Gcn1(-Gcn20)-Rbg2-Gir2-80S) and the colliding ribosome (Gcn1-Mbf1-80S) have been deposited in the Electron Microscopy Data Bank with the accession codes EMD-12534 and EMD-12535. The associated molecular models of the leading (Gcn1-Rbg2-Gir2-80S) and the colliding (Mbf1-80S) ribosome are available in the Protein Data Bank with the accession PDB ID 7NRC and 7NRD, respectively.

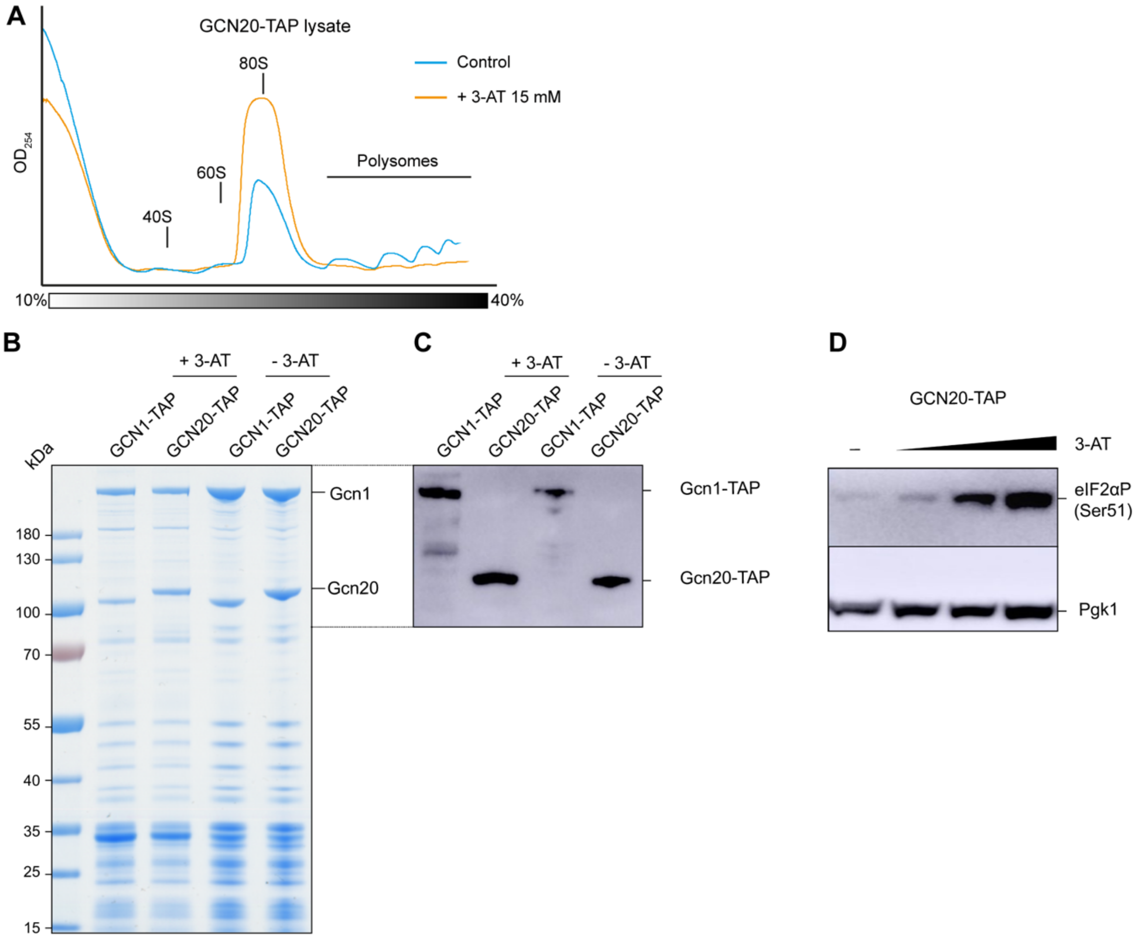

Fig. S1 Analysis of the pull-outs of the Gcn1- and Gcn20 C-terminally TAP-tagged samples. (**A**) GCN20-TAP whole cell extracts cultured with 15 mM (orange trace) or without (blue trace) 3-AT were subjected to sucrose density gradient centrifugation. Gradients were fractionated while scanning at 254 nm and the resulting absorbance profiles are shown. Positions of 40S, 60S, 80S and polysomes are indicated. (**B**) 4-12% NuPAGE gel of the Gcn1- and Gcn20-TAP-tag purified complexes from the cell cultures in presence or absence of 15 mM 3-AT. (**C**) as in (**B**), but analyzed via SDS-PAGE and immunoblotting using a TAP-tag polyclonal antibody. (**D**) Phosphorylation level of eIF2α analyzed by Western blotting. Whole cell extracts of the GCN20-TAP strain were prepared from exponential growing cell cultures treated with 1.5 and 15 mM 3-AT. The phosphorylation level of eIF2α was then monitored by immunoblotting, while immunoblotting against PGK1 was used as loading control.

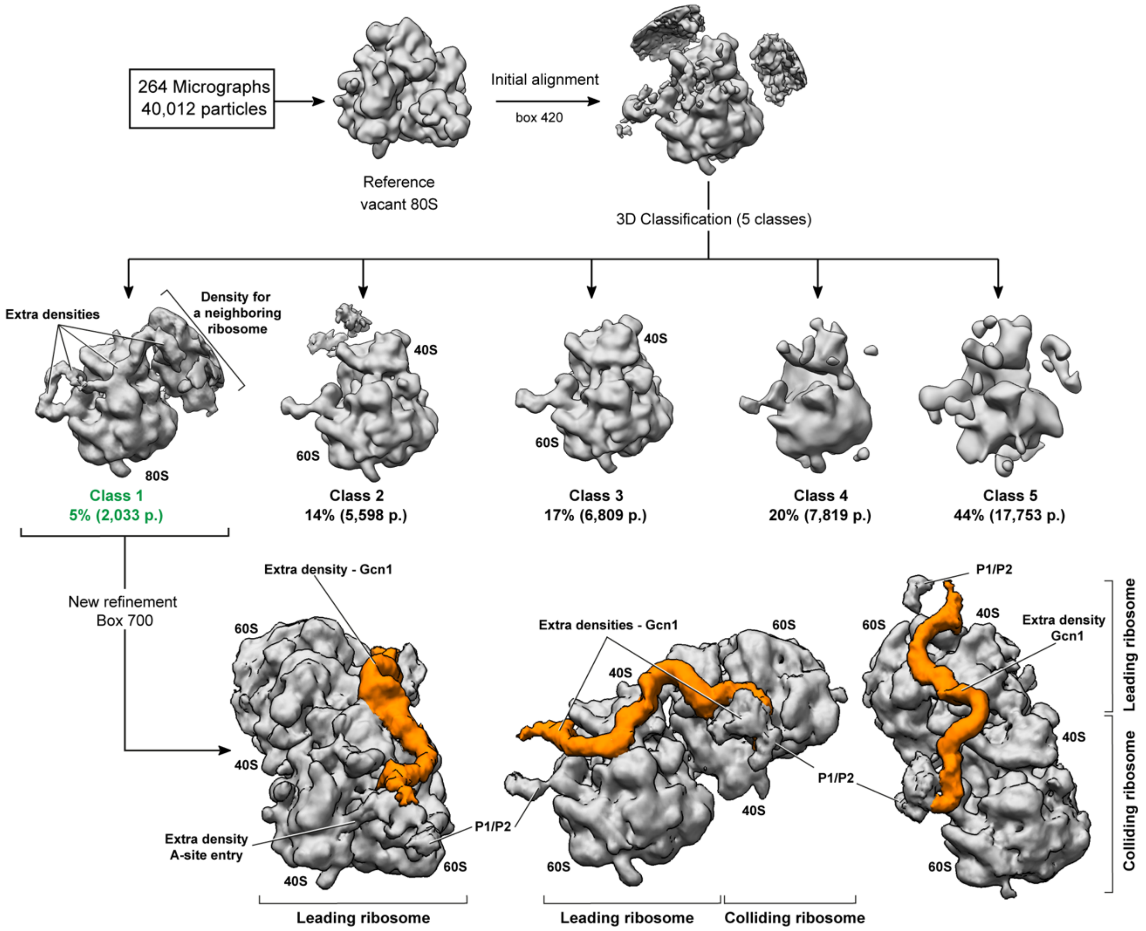

Fig. S2. Processing of the low resolution Gcn20-TAP dataset. After automatic acquisition of 264 micrographs, 40,012 particles were selected for the initial reconstruction with an initial alignment using a vacant *S. cerevisiae* 80S ribosome. The particles were then subjected to 3D classification, sorting the particles into 5 classes. Class 2 and 3 contain vacant ribosomes without any extra density at the nominal resolution of 21 Å. Class 1 with the extra densities was then refined using a bigger box size and shows a disome with an extra density connecting the two ribosomes (orange) as well as an extra density at the A-site entry.

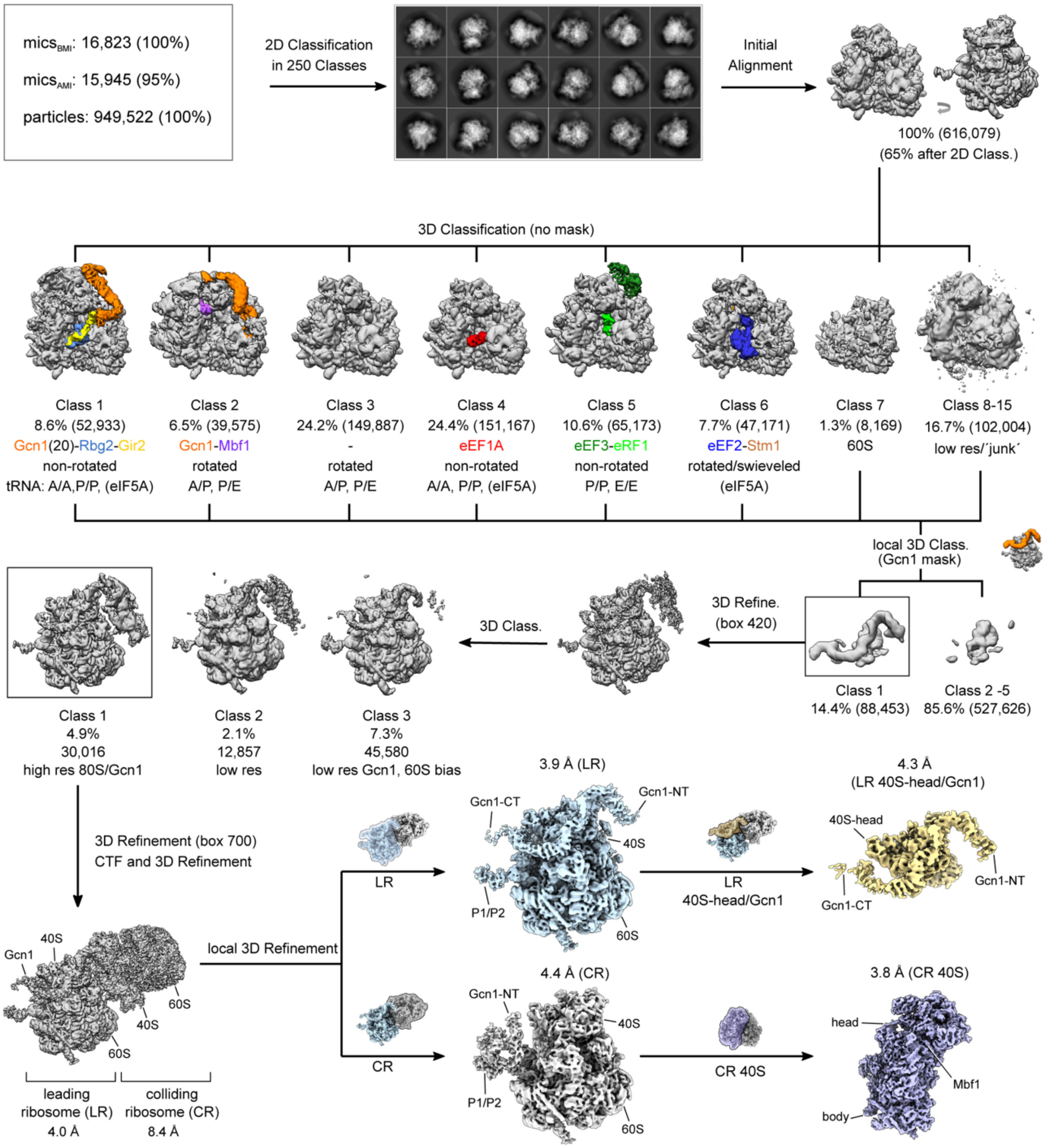

Fig. S3. Processing of the high resolution Gcn20-TAP dataset. After manual inspection (AMI; before manual inspection, BMI) of 16,823 micrographs, 15,945 were selected for the initial particle picking, resulting in 949,522 particles. Following 2D classification, 616,079 particles were initially aligned against a vacant *S. cerevisiae* 80S ribosome and subjected to 3D classification, sorting the particles into 15 initial classes as depicted. Subsequently, all classes (1-15) were re-combined and subjected to local sorting using a mask for Gcn1. Class 1 (14.3%, 88,453 particles) containing Gcn1 bound particles was subsequently refined and 3D classified into three classes. High resolution particles in Class1 (4.9%, 30,016 particles) were again 3D and CTF refined with an enlarged box size and resulted in a final disome reconstruction at 4.0 Å for the leading ribosome (LR) and 8.4 Å for the colliding ribosome (CR). The LR (blue) and CR (grey) were masked and individually local refined resulting to 3.9 Å and 4.4 Å, respectively. To increase the resolution of Gcn1 and Mbf1, the LR and CR were subjected to an further local refinement with an individual mask encompassing the important regions: the 40S head and Gcn1 (yellow mask) for the LR and 40S (purple mask) for the CR, which resulted in final reconstructions of the masked regions at a final resolution of 4.3 Å and 3.8 Å, respectively.

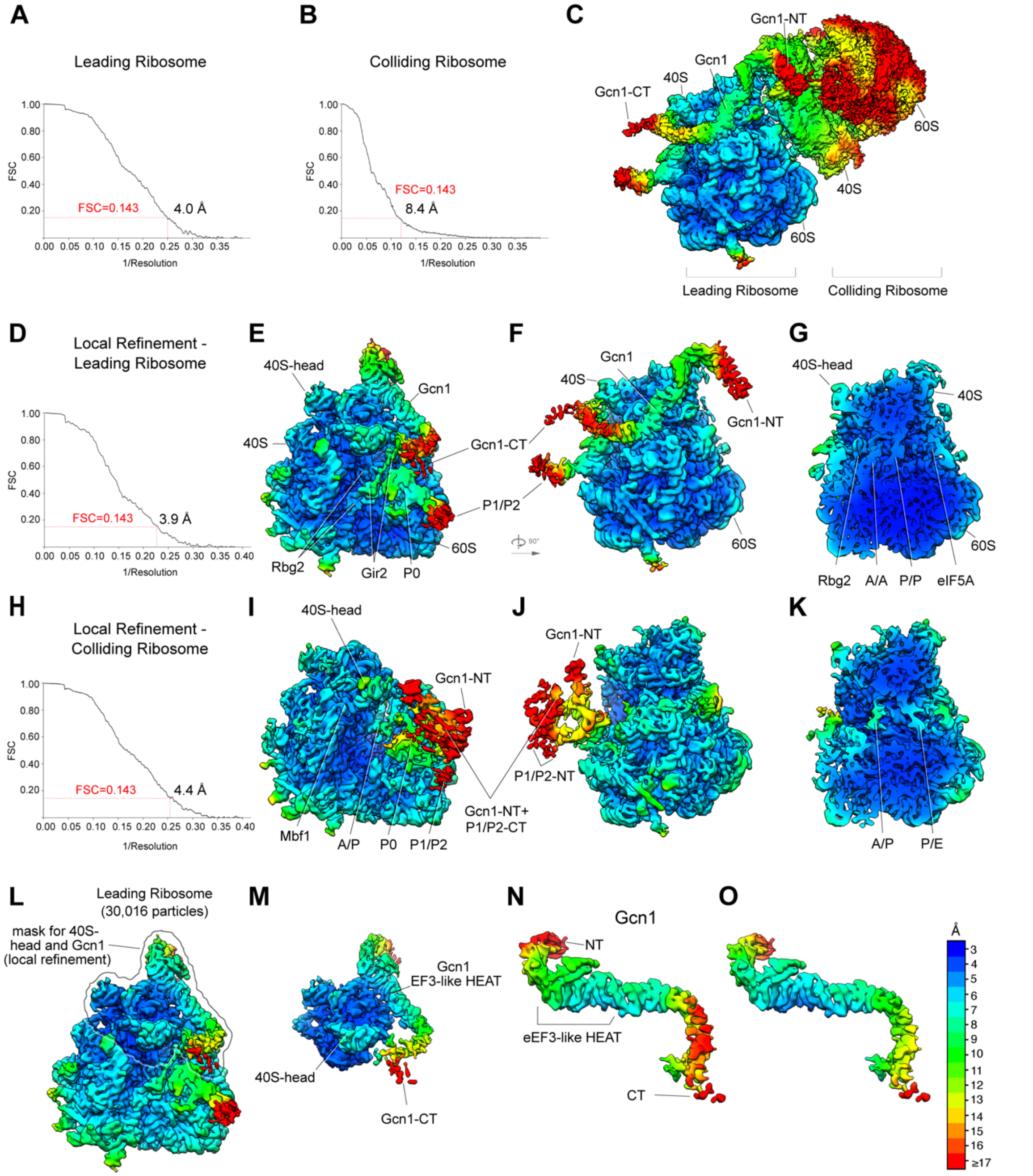

Fig. S4. Local resolution of cryo-EM reconstructions of the Gcn1-disome. (**A-C**) Cryo-EM reconstruction of the Gcn1-disome, with (**A-B**) Fourier shell correlation (FSC) curve of the final reconstructions indicating an average resolution of 4.0 Å for the leading and 8.4 Å for the colliding ribosome, according to the gold-standard criterion (FSC=0.143). (**C**) Cryo-EM map of the full disome filtered to 8 Å and colored according to local resolution. (**D-G**) Cryo-EM reconstruction of the leading ribosome, with (**D**) FSC curve of the final reconstruction indicating an average resolution of 3.9 Å (FSC=0.143). (**E,F**) Cryo-EM reconstruction of the leading ribosome filtered to 8 Å and colored according to the local resolution. (**G**) Transverse section of the volume shown in (**F**). (**H-K**) Cryo-EM reconstruction of the colliding ribosome, with (**H**) FSC curve of the final reconstruction indicating an average resolution of 4.4 Å (FSC=0.143). (**I,J**) Cryo-EM reconstruction of the leading ribosome filtered to 9 Å and colored according to the local resolution. (**K**) Transverse section of the

volume shown in (**J**). (**L**) Cryo-EM reconstruction of the locally refined leading ribosome colored according to local resolution. The border represents the mask, which was used for the local refinement of Gcn1 including Gcn1 and the head of the 40S subunit of the leading ribosome. (**M**) Postprocessed volume shown in (**L**). (**N-O**) Local resolution of Gcn1 (**N**) before and (**O**) after the local refinement.

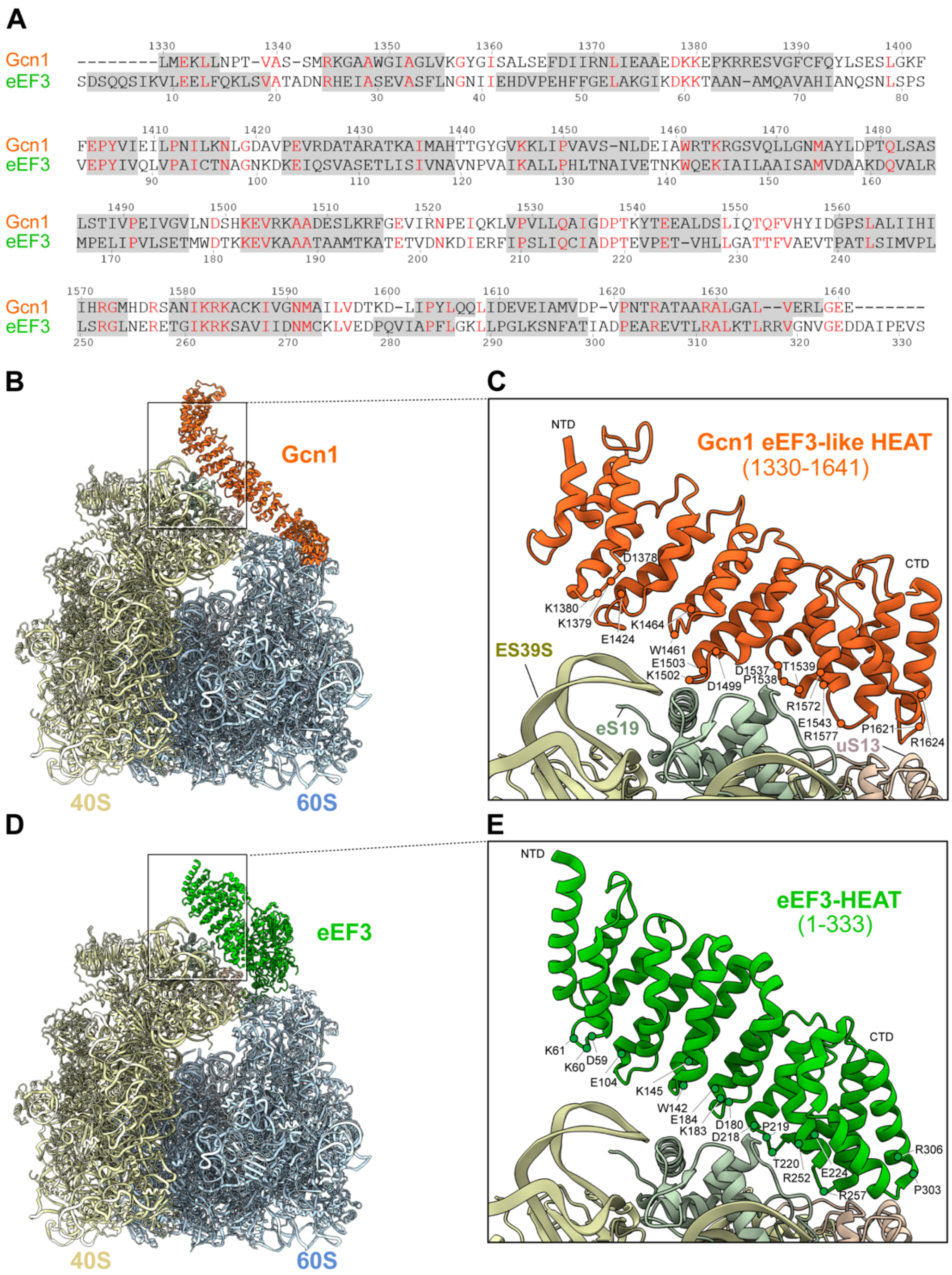

Fig. S5. Comparison between the Gcn1 eEF3-like HEAT region and eEF3 HEAT repeats. (**A**) Sequence alignment of the eEF3-like HEAT repeat region of Gcn1 with HEAT repeats in eEF3. Conserved residues are denoted in red and helices are marked as grey boxes. (**B**) Molecular model of the Gcn1-80S complex (Gcn1, orange; 40S, pale yellow; 60S, cyan) with (**C**) zoomed view showing the detailed amino acid composition of the Gcn1 region interacting with ES39S (pale yellow), eS19 (pale green), uS13 (pale coral) of the 40S on the leading ribosome. (**D**) The eEF3 ribosomal model (eEF3, green) with (**E**) enlarged view of the eEF3 interface contacting the ribosome.

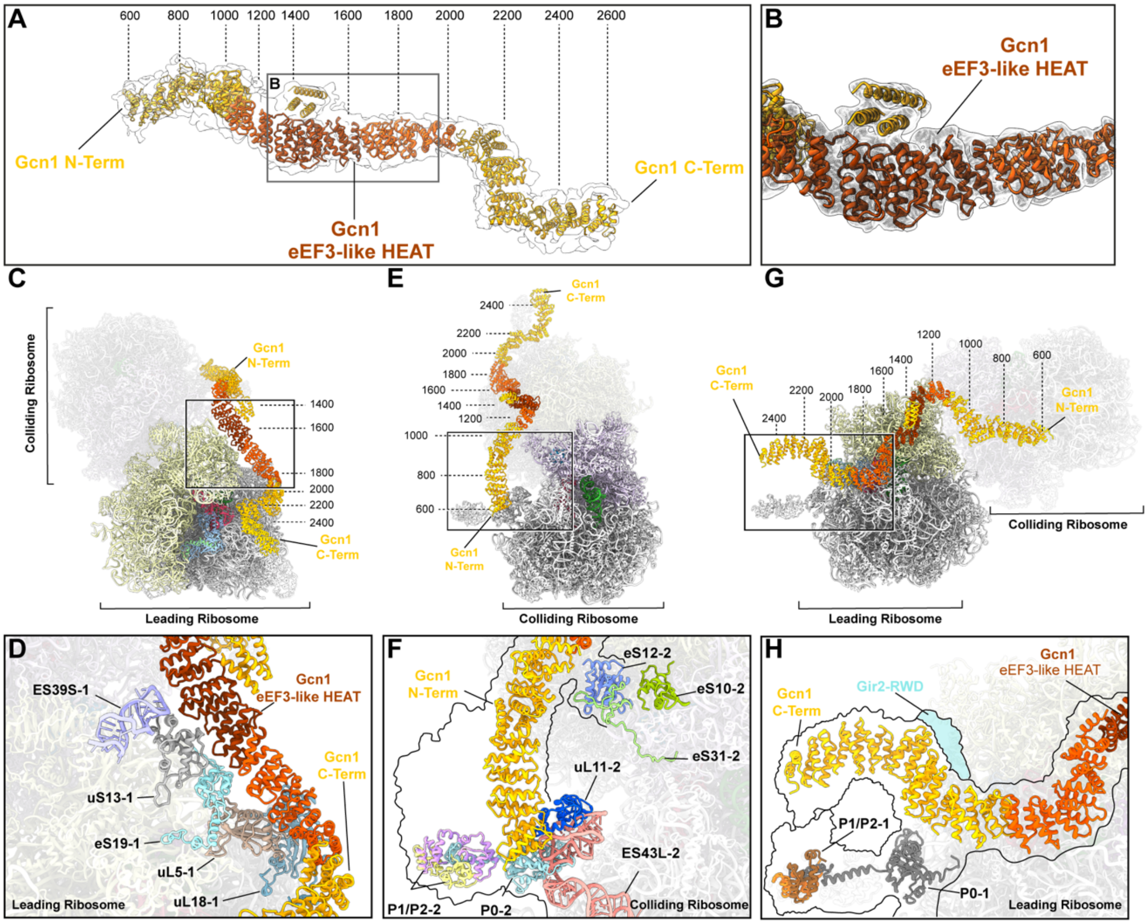

Fig. S6. Interactions of Gcn1 within the disome. (**A**) Molecular model of the Gcn1 within the cryo-EM density (gray transparent). The eEF3-like region (dark orange) is shown enlarged in (**B**). The bright orange HEAT repeats were fitted individually based on the features of the density for helices, whereas the yellow HEAT repeats are tentative placements fitted to the map to obtain an approximate estimation of the HEAT positions within Gcn1. (**C-H**) Interactions of Gcn1 within the disome, specifically focused on the (**C-D**) central region of Gcn1 interacting with ES39S, uS13 and eS19 of the 40S head and uL5 and uL18 of the central protuberance of the leading ribosome, (**E-F**) N-terminal region of Gcn1 interacting with uL11, ES43L and the P-stalk proteins P0, P1 and P2 of the colliding ribosome, and (**G-H**) the C-terminal region of Gcn1 interacting with the RWD domain of Gir2 (cyan) as well as the P-stalk proteins P0, P1 and P2 of the leading ribosome. The border shown in (**F**) and (**H**) reflects the density within the cryo-EM map.

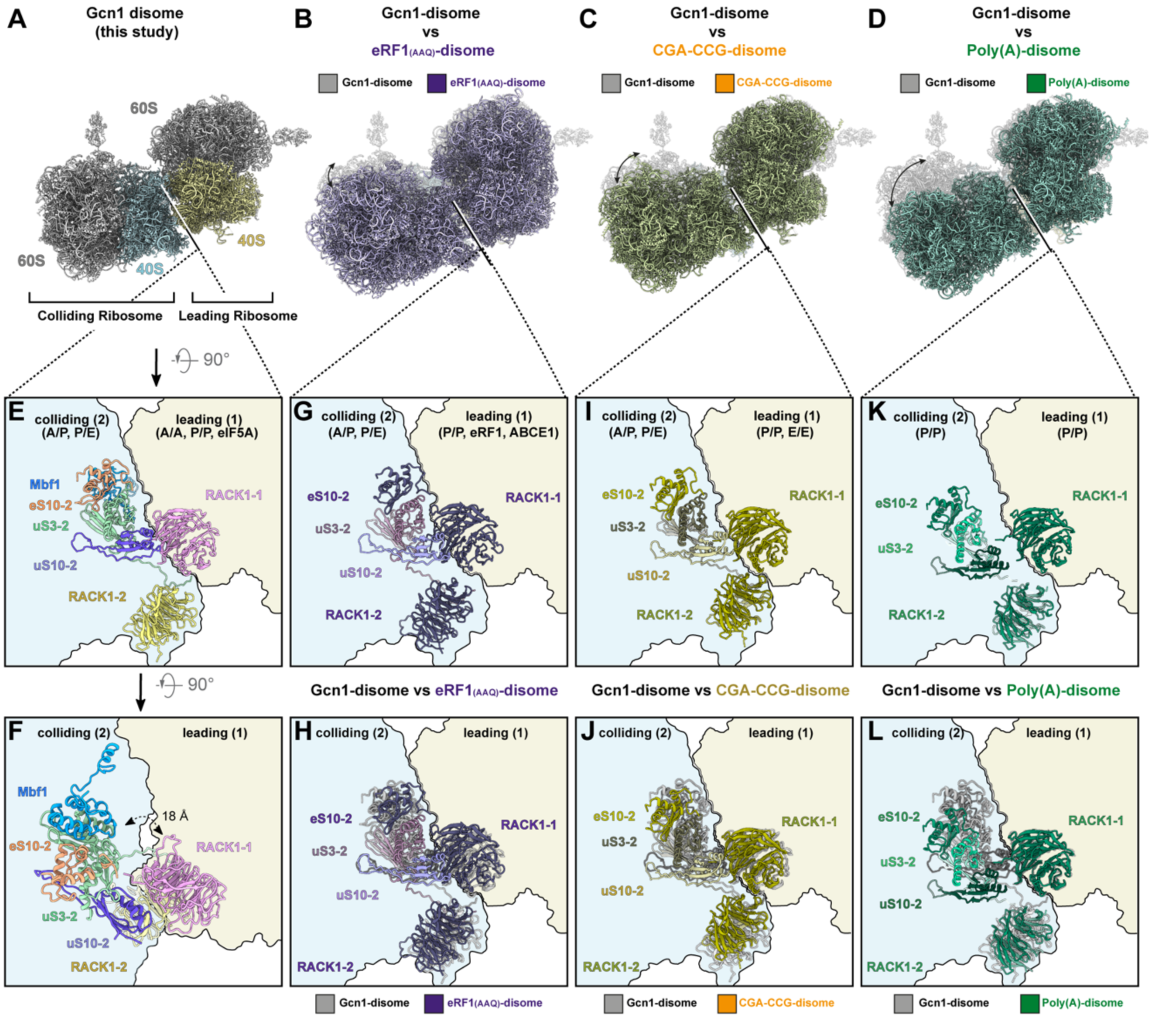

Fig. S7. 40S-40S interaction interface of the Gcn1-bound disome and its comparison to other disomes. (**A**) Overview of the Gcn1-disome molecular model. (**B**) Collided disome stalled by a catalytically nonactive eRF1 mutant (eRF1-AAQ) (PDB ID: 6hcm, 6hcq) (22) (purple) overlaid with the Gcn1-disome (grey) shown in (**A**). (**C**) Disome stalled on a CGA-CCG mRNA (PDB ID: 6i7o) (23) (orange) overlaid with the Gcn1-disome (grey) structure shown in (**A**). (**D**) Collided disome stalled on poly(A) mRNA (PDB ID: 6t83) (24) (dark green) overlaid with the Gcn1-disome (grey) structure shown in (**A**). (**E, F**) Close-up view on the Gcn1-disome interaction interface of the 40S subunits from the leading stalled ribosome (pale yellow) and the colliding ribosome (pale blue). (**G**) Close-up view on the interaction interface from the collided disome stalled by a eRF1-AAQ mutant. (**H**) Overlay of (**E**) and (**G**). (**I**) Close-up view on the interaction interface from the disome stalled on a CGA-CCG mRNA. (**J**) Overlay of (**E**) and (**I**). (**K**) Close-up view on the interaction interface from the collided disome stalled on poly(A) mRNA. (**L**) Overlay of (**E**) and (**K**).

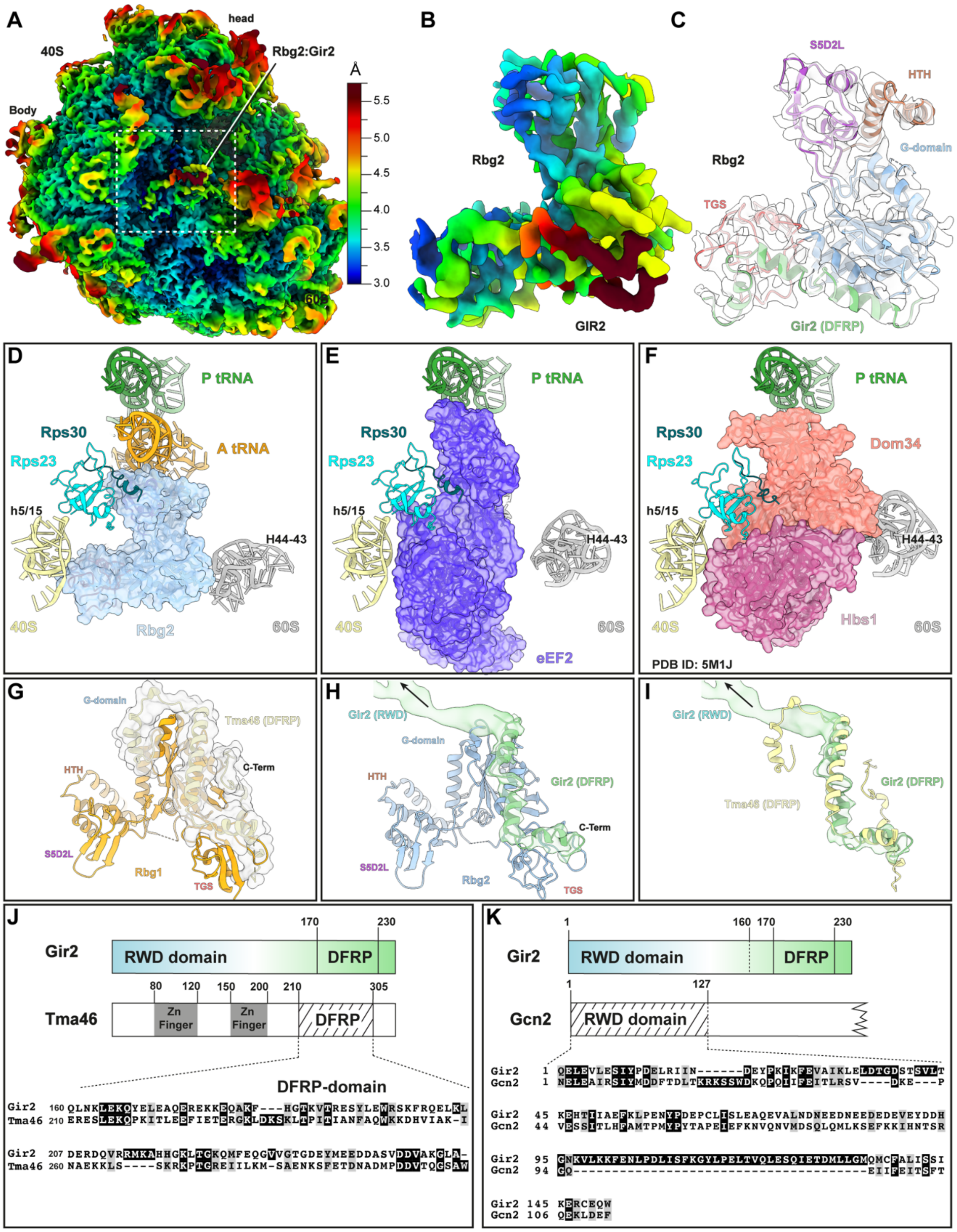

Fig. S8. Molecular model for Rbg2 and comparison of Gir2 with Tma46 and Gcn2. (**A**) Local resolution map of the leading ribosome with the binding site of Rbg2 indicated. (**B**) Isolated density of the Rbg2-Gir2 complex from the Gcn1-disome, coloured according to local resolution. (**C**) Molecular model for Rbg2 fitted into isolated cryo-EM density for Rbg2 (transparent grey) and colored by domain. (**D-F**) Comparison of (**D**) Rbg2 (cyan) and A-tRNA (orange) with (**E**) eEF2 (blue, PDB ID 6GQV) (25) and (**F**) Hbs1 (pink) and Dom34 (salmon, PDB ID 5M1J) (26). (**G**) Structure of Rbg1 (orange, PDB ID: 4A9A) (17) in complex with Tma46 (DFRP) domain (light yellow with transparent surface). (**H**) Conformation of Rbg2 (blue) and Gir2(DFRP) domain (green with

transparent surface) as found bound to a stalled leading ribosome in the Gcn1-disome. (**I**) comparison of cryo-EM density and model for Gir2 (green) with the model for Tma46 as obtained by aligning the Rbg1-Tma46 complex to Rbg2. (**J**) Schematic representation of the domain structure of Gir2 and Tma46 with zoom on a sequence alignment of their respective DFRP domains. (**K**) Schematic representation of the domain structure of Gir2 and Gcn2 with a zoom on a sequence alignment their respective RWD domains.

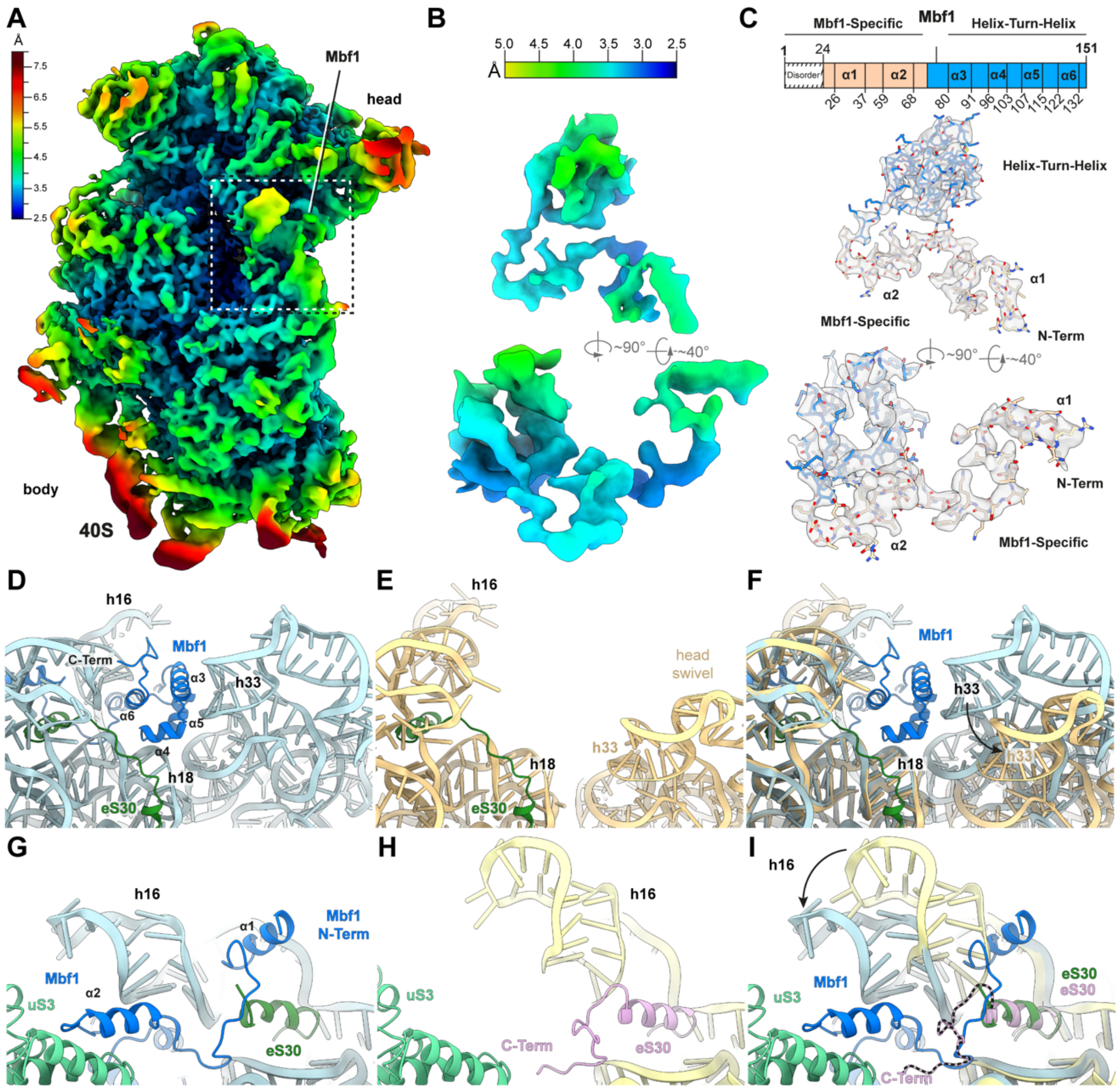

Fig. S9. Molecular model for Mbf1 on the Gcn1-disome. (**A**) Local resolution map of the 40S subunit of the colliding ribosome from the Gcn1-disome with the binding site of Mbf1 indicated. (**B**) Two views of the isolated density of the Mbf1 colored according to local resolution. (**C**) Schematic representation of Mbf1 showing the Mbf1-specific N-terminus (light brown, residues 44-79) and the C-terminal helix-turn-helix domain (blue). Isolated density (grey transparency) with fitted molecular model for Mbf1 (colored by domain as illustrated in the scheme). (**D**) Mbf1 C-terminal helix-turn-helix interacting with the 18S rRNA. (**E**) Head swivel 40S ribosome (yellow). (**F**) Similar view to (**D** and **E**) with an overlay of head swivel 40S ribosome (yellow). (**G-I**) Conformation change of eS30 C-terminal region and h16 in the Mbf1 bound structure compared to a reference structure (PDB ID 6SNT, pink for eS30 and yellow for h16).

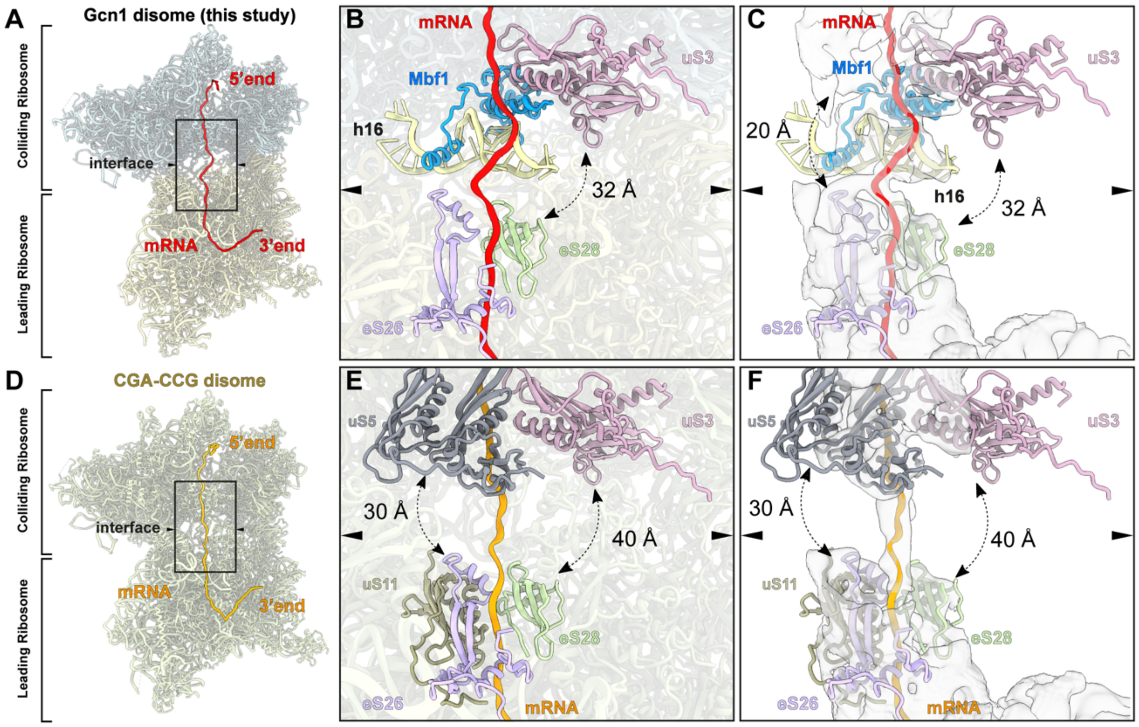

Fig. S10. Compaction of the Gcn1-disome. (**a**) 40S of the leading (pale yellow) and the colliding (cyan) ribosome of the Gcn1-stalled disome containing the mRNA (red). (**b**) Close-up view of the 40S-40S interface shown in (**a**). The ribosomal proteins and rRNA interacting with the mRNA are depicted and labelled. (**c**) View as in (**b**) including the locally isolated density for the mRNA and the surrounding ribosomal components. (**d**) 40S subunits (tan) of the disome stalled on CGA-CCG mRNA (PDB ID: 6I7O) (23) (mRNA in light orange) and its (**e**) enlarged view of the 40S-40S interface (**f**) including the locally isolated density for the mRNA and the surrounding ribosomal proteins.

Table S1 | Cryo-EM data collection, refinement and validation statistics.

	Leading stalled ribosome<br>EMD ID 12534<br>PDB ID 7NRC	Colliding ribosome<br>EMD ID 12535<br>PDB ID 7NRD
**Data collection**		
Microscope	FEI Titan Krios	FEI Titan Krios
Camera	Falcon II	Falcon II
Magnification	131,703	131,703
Voltage (kV)	300	300
Electron dose (e^{-}/Å^2)	25	25
Defocus range (μm)	-1.3 to -2.8	-1.3 to -2.8
Pixel size (Å)	1.084	1.084
Initial particles (no.)	616,079	616,079
Final particles (no.)	30,016	30,016
Map resolution (Å)	3.85	4.36
Box size	700,700,700	700,700,700
**Model composition**		
Protein residues	11829	11163
RNA bases	5391	5430
**Refinement**		
Resolution range (Å)	3.0-15	3.3-15
Map CC (around atoms)	0.74	0.74
Map sharpening *B* factor (Å^2)	-84.4315	-120.184
R.m.s. deviations		
Bond lengths (Å) (#>4σ)	0.006	0.012
Bond angles (°) (#>4σ)	1.311	1.384
**Validation**		
MolProbity score	1.76	2.06
Clashscore	4.43	9.22
Poor rotamers (%)	0.27	1.21
**Ramachandran plot**		
Favored (%)	90.44	89.29
Allowed (%)	9.46	10.50
Disallowed (%)	0.09	0.16

Movie S1 (separate file). Overview of Gcn1 and interactions within colliding disome structure. Overview of the model of Gcn1 (orange), then zoom out to show electron density for disome and Gcn1 and Gcn20. Rotations to highlight factor in complex with leading ribosome including A-tRNA (red), P-tRNA (green), Rbg2 (blue) and Gir2 (green), as well as in colliding ribosome with Mbf1 (blue), A/P-tRNA (tan) and P/E-tRNA (pink).

Dataset S1 (separate file). Mass spectrometry analysis of Gcn20 affinity purification samples.

SI References

1. Schmidt C*, et al.* (2016) The cryo-EM structure of a ribosome-Ski2-Ski3-Ski8 helicase complex. *Science* 354(6318):1431-1433.
2. Ranjan N*, et al.* (2021) Yeast translation elongation factor eEF3 promotes late stages of tRNA translocation. *EMBO J*:e106449.
3. Tyanova S, Temu T, & Cox J (2016) The MaxQuant computational platform for mass spectrometry-based shotgun proteomics. *Nat Protoc* 11(12):2301-2319.
4. Schwanhausser B*, et al.* (2011) Global quantification of mammalian gene expression control. *Nature* 473(7347):337-342.
5. Elias JE & Gygi SP (2007) Target-decoy search strategy for increased confidence in large-scale protein identifications by mass spectrometry. *Nature methods* 4(3):207-214.
6. Zheng SQ*, et al.* (2017) MotionCor2: anisotropic correction of beam-induced motion for improved cryo-electron microscopy. *Nature methods* 14(4):331-332.
7. Zhang K (2016) Gctf: Real-time CTF determination and correction. *Journal of structural biology* 193(1):1-12.
8. Zivanov J*, et al.* (2018) New tools for automated high-resolution cryo-EM structure determination in RELION-3. *eLife* 7.
9. Svidritskiy E, Brilot AF, Koh CS, Grigorieff N, & Korostelev AA (2014) Structures of yeast 80S ribosome-tRNA complexes in the rotated and nonrotated conformations. *Structure* 22(8):1210-1218.
10. Behrmann E*, et al.* (2015) Structural snapshots of actively translating human ribosomes. *Cell* 161(4):845-857.
11. Pittman YR*, et al.* (2006) Mg2+ and a key lysine modulate exchange activity of eukaryotic translation elongation factor 1B alpha. *The Journal of biological chemistry* 281(28):19457-19468.
12. Anger AM*, et al.* (2013) Structures of the human and Drosophila 80S ribosome. *Nature* 497(7447):80-85.
13. Brown A, Baird MR, Yip MC, Murray J, & Shao S (2018) Structures of translationally inactive mammalian ribosomes. *eLife* 7.
14. Bienert S*, et al.* (2017) The SWISS-MODEL Repository-new features and functionality. *Nucleic acids research* 45(D1):D313-D319.

15. Pettersen EF*, et al.* (2004) UCSF Chimera--a visualization system for exploratory research and analysis. *Journal of computational chemistry* 25(13):1605-1612.
16. Emsley P & Cowtan K (2004) Coot: model-building tools for molecular graphics. *Acta crystallographica. Section D, Biological crystallography* 60(Pt 12 Pt 1):2126-2132.
17. Francis SM, Gas ME, Daugeron MC, Bravo J, & Seraphin B (2012) Rbg1-Tma46 dimer structure reveals new functional domains and their role in polysome recruitment. *Nucleic acids research* 40(21):11100-11114.
18. Croll TI (2018) ISOLDE: a physically realistic environment for model building into low-resolution electron-density maps. *Acta Crystallogr D Struct Biol* 74(Pt 6):519-530.
19. Adams PD*, et al.* (2010) PHENIX: a comprehensive Python-based system for macromolecular structure solution. *Acta crystallographica. Section D, Biological crystallography* 66(Pt 2):213-221.
20. Schmidt C*, et al.* (2016) Structure of the hypusinylated eukaryotic translation factor eIF-5A bound to the ribosome. *Nucleic acids research* 44(4):1944-1951.
21. Goddard TD*, et al.* (2018) UCSF ChimeraX: Meeting modern challenges in visualization and analysis. *Protein science : a publication of the Protein Society* 27(1):14-25.
22. Juszkiewicz S*, et al.* (2018) ZNF598 Is a Quality Control Sensor of Collided Ribosomes. *Molecular cell* 72(3):469-481 e467.
23. Ikeuchi K*, et al.* (2019) Collided ribosomes form a unique structural interface to induce Hel2-driven quality control pathways. *EMBO J* 38(5).
24. Tesina P*, et al.* (2020) Molecular mechanism of translational stalling by inhibitory codon combinations and poly(A) tracts. *EMBO J* 39(3):e103365.
25. Pellegrino S*, et al.* (2018) Structural Insights into the Role of Diphthamide on Elongation Factor 2 in mRNA Reading-Frame Maintenance. *J Mol Biol* 430(17):2677-2687.
26. Hilal T*, et al.* (2016) Structural insights into ribosomal rescue by Dom34 and Hbs1 at near-atomic resolution. *Nat Commun* 7:13521.
